# Sex-related differences in the clinical presentation of multiple system atrophy

**DOI:** 10.1007/s10286-024-01028-1

**Published:** 2024-04-17

**Authors:** Fabian Leys, Sabine Eschlböck, Nicole Campese, Philipp Mahlknecht, Marina Peball, Georg Goebel, Victoria Sidoroff, Florian Krismer, Roberta Granata, Stefan Kiechl, Werner Poewe, Klaus Seppi, Gregor K. Wenning, Alessandra Fanciulli

**Affiliations:** 1 Department of Neurology, Medical University of Innsbruck, Innsbruck, Austria; 2 Department of Neurology, Hochzirl-Natters Hospital, Zirl, Austria; 3 Institute of Medical Statistics and Informatics, Medical University of Innsbruck, Innsbruck, Austria; 4 Department of Neurology, Provincial Hospital of Kufstein, Kufstein, Austria

**Keywords:** Multiple system atrophy, Sex, Gender, Differences, Clinical presentation

## Abstract

**Purpose:**

To investigate sex-related differences in the clinical presentation of multiple system atrophy (MSA) through a literature review and an analysis of a retrospective cohort.

**Methods:**

The PubMed database was searched for articles including sex-related information in MSA. In a retrospective Innsbruck cohort, we investigated the baseline to last available follow-up clinical-demographic differences between men and women with MSA in a univariate fashion, followed by multivariable binary regression analysis.

**Results:**

The literature search yielded 46 publications with sex-related information in MSA. Most studies found comparable survival rates between the sexes, while some recent reports suggested a potential survival benefit for women, possibly due to initial motor onset and overall less severe autonomic failure compared to men. The retrospective Innsbruck MSA cohort comprised 56 female and 60 male individuals with a comparable median follow-up of 27 months. At baseline, female sex was independently associated with depression (odds ratio [OR] 4.7; *p* = 0.007) and male sex with severe orthostatic hypotension (OR 5.5; *p* = 0.016). In addition, at last follow-up, female sex was associated with the intake of central nervous system-active drugs (OR 4.1; *p* = 0.029), whereas male sex was associated with the presence of supine hypertension (OR 3.0; *p* = 0.020) and the intake of antihypertensive medications (OR 8.7; *p* = 0.001). Male sex was also associated with initiation of antihypertensive medications over the observation period (OR 12.4; *p* = 0.004).

**Conclusion:**

The available literature and findings of the present study indicate sex-related differences in the clinical presentation of MSA and its evolution over time, highlighting the importance of considering sex in symptom exploration, therapeutic decision-making, and future clinical trial design.

**Supplementary Information:**

The online version contains supplementary material available at 10.1007/s10286-024-01028-1.

## Introduction

Multiple system atrophy (MSA) is a rare, rapidly progressive, and fatal neurodegenerative disorder of the adulthood, characterized by severe, multi-domain autonomic failure, poorly levodopa (L-Dopa)-responsive parkinsonism, and cerebellar and pyramidal features in various combinations [1]. Depending on the predominant motor presentation, a Parkinsonian (MSA-P) and a cerebellar variant (MSA-C) are distinguished [1]. Phosphorylated α-synuclein glial cytoplasmic inclusions associated with striatonigral, olivopontocerebellar, and central autonomic degeneration constitute the neuropathological hallmark of MSA and differentiate it from Parkinson’s disease (PD), in which neuronal α-synuclein aggregates—the so-called Lewy bodies—accompany the neurodegenerative process [2, 3].

The term “sex” refers to the distinction of human individuals as female or male according to their reproductive organs and functions that derive from the chromosomal complement and determine differences in anatomy, physiology, and hormones [4]. By contrast, “gender” describes a person’s self-representative identity, which may most commonly be male or female, but also encompasses transgender and other non-binary identities, and influences social interactions by the norms, behaviors, and roles associated with identifying a person as female, male, or non-binary [4].

Over the past years, both sex- and gender-related differences have been increasingly acknowledged as important factors influencing the clinical management and research outcomes of several neurodegenerative diseases [5]. In PD, differences between men and women have been found in the natural history, clinical profile, therapeutic needs, and treatment complications. The incidence and prevalence of PD is on average lower in women, who tend to be older at disease onset, suffer more frequently from disabling tremor, dyskinesia, and both motor and non-motor fluctuations, and have less access to deep brain stimulation compared to men [6]. Gender is also known to influence daily living and the care pathways of people with PD at multiple levels [6, 7].

By contrast, the incidence of MSA is comparable between women and men, and no clear sex-dependent difference has been identified so far in disease progression or survival of individuals living with MSA [1, 8]. Sex-related aspects have recently gained attention in MSA settings as well [9], but only a limited number of studies have specifically addressed this matter to date [10–12], and it ultimately remains unclear whether sex has an influence on MSA clinical presentation or its evolution over time.

In the study reported here, we first aimed to perform a narrative literature review on sex-related differences in the clinical presentation and natural history of MSA. Subsequently, we investigated whether sex-related differences occurred in the symptomatic profile or emerged over time in a large, retrospective cohort of individuals with MSA.

## Methods

### Literature search

**Fig. 1:**
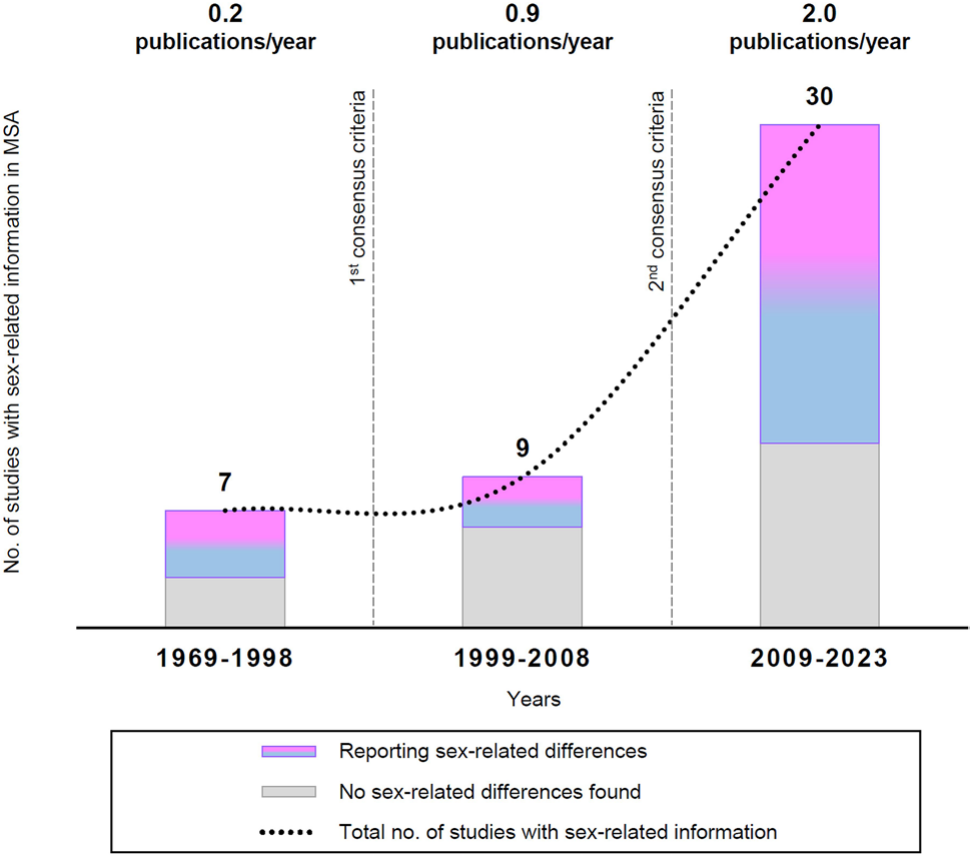
Studies providing sex-related information in the clinical presentation or natural history of MSA from its first description in 1969 through 2023. * MSA* multiple system atrophy

Articles including sex-related information in the clinical presentation and natural history of MSA were identified by means of a PubMed literature search using the following combination of keywords: [“multiple system atrophy”] *AND* [“gender”] *OR* [“sex”] *OR* [“women”] *OR* [“men”]. The titles and abstracts of all search hits were screened for pertinence and relevance and, whenever applicable, a full-text review was subsequently performed. The references of selected articles and relevant publications known to the study team were counterchecked for additional eligible papers. Records in English language published between 1969, when the term “multiple system atrophy” was first introduced in the scientific literature from Graham and Oppenheimer [13], through June 2023 were considered. A schematic overview of the literature search is provided in Electronic Supplementary Material (ESM) Fig. 1.

### Study population and data collection

The present study population comprised individuals retrospectively included in the Innsbruck MSA Registry who: (1) at the last available follow-up fulfilled the second consensus criteria for the diagnosis of MSA [14], namely probable MSA *OR* possible MSA with ≥ 3 years disease duration; and (2) had a baseline visit and at least one follow-up visit.

All patients were examined at the Department of Neurology of the Medical University of Innsbruck, Austria, between 1998 and 2020. Clinical-demographic information concerning the baseline and last follow-up visit was retrospectively collected from available electronic and hand-written medical records and included:

- demographic data (i.e., sex; age at onset, baseline visit, and last available follow-up; disease duration to baseline visit and last available follow-up; and follow-up time);
- initial clinical features (divided into a motor, autonomic, or combined motor and autonomic symptomatic onset);
- presence of MSA motor or non-motor features and their associated treatment, including, among others:
  - classic orthostatic hypotension (OH), defined as orthostatic blood pressure (BP) falls of ≥ 20 mmHg systolic BP or ≥ 10 mmHg diastolic BP within 3 min of active or passive orthostatic challenge [15];
  - severe OH, defined as history of recurrent syncope and orthostatic BP falls ≥ 30 mmHg systolic BP or ≥ 15 mmHg diastolic BP [15];
  - intake of central nervous system (CNS)-active drugs with BP-lowering side effects, including anti-depressants, benzodiazepines, anti-psychotics, opioids, and trazodone [16];
- rating scales (Hoehn and Yahr stage [17] and Unified MSA Rating Scale [UMSARS] Part IV score [18]);
- comorbidities (presence of any cardio- and/or cerebrovascular disease, or diabetes mellitus).

A detailed description of the clinical-demographic information retrieval strategy and definitions of variables is provided in ESM Table 1.

### Statistical analysis

Qualitative variables were summarized by absolute frequency (percentage) and quantitative variables were summarized by the median [25th; 75th percentile]. Absent information was treated as “missing data”. The available sample size for each given analysis is reported in the tables in parentheses (*n*), and the relative frequencies correspond to the proportion of available data. Qualitative variables were analyzed using Pearson’s Chi-square (*χ*^2^), Fisher’s exact, or Fisher-Freeman-Halton test, where appropriate. The Kolmogorov–Smirnov test was used to test the distribution of quantitative variables. Depending on the data distribution, differences in quantitative variables were assessed with the Student’s *t*-test or Mann–Whitney *U*-test, and bivariate correlations coefficients were calculated according to Pearson’s or Spearman’s *ρ*. Binary logistic regression analysis was used to create multivariable regressions models. The Hosmer–Lemeshow test was used to compare observed and expected event rates of logistic regressions models.

As the first step, we performed a univariate analysis to investigate the differences in demographic data, initial clinical features, and the clinical presentation at baseline and last available follow-up of female versus male individuals with MSA. We then calculated the change in clinical characteristics (including new onset of motor/non-motor MSA features and comorbidities; start of any associated treatment; progression in the Hoehn and Yahr stage or dependency with core activities [i.e., UMSARS Part IV]; adjustment of L-dopa equivalent daily dosage; and worsening of dopaminergic response) from baseline to last follow-up for each individual and compared the respective rate of change from baseline to last follow-up between sexes. In a second step, clinical characteristics with a *p* value of < 0.1 in the univariate comparison between female and male MSA individuals were selected as dependent outcome variables for the multivariable binary regression analyses to investigate possible associations with sex and other covariates. In addition to sex, other covariates included in each multivariable model were age, disease duration or follow-up time, and other clinically meaningful parameters, depending on the outcome variable of interest (e.g., anti-hypertensive medication intake for the outcome variable “severe OH”). Taking the cohort size into account, we allowed up to a maximum of six covariates without high direct (i.e., Pearson’s/Spearman’s *ρ* ≥ 0.7) or inverse (i.e., Pearson’s/Spearman’s *ρ* ≤ − 0.7) bivariate correlations for each multivariable analysis. Whenever the calculation of multivariable odds ratios was not feasible due to high interference of covariates included in the model (e.g., covariates which were present in most of the subjects), bivariate odds ratios were presented.

A two-tailed *p* value of < 0.05 was considered to be statistically significant. Due to the study’s explorative nature, we did not apply a correction for multiple testing. The statistical analysis was performed with the SPSS® V29.0 statistical package (SPSS IBM Corp., Armonk, NY, USA). The full analytic approach of the present study is summarized in ESM Fig. 2.

## Results

### Literature review on sex-related differences in the clinical presentation and natural history of MSA

The literature search yielded 46 articles that included information on sex-related differences in the clinical presentation and natural history of MSA. Figure 1 depicts the number of studies reporting sex-related information before [19–25] and after [26–34] the publication of the first [35] and second MSA consensus criteria [14] through June 2023 [10–12, 36–62].

Most studies comprised clinically diagnosed MSA cohorts, while six studies included post-mortem MSA cases only [21, 24, 31, 32, 34, 42]. Both clinical and neuropathological studies on MSA found sex-related differences in survival and clinical presentation of the disease.

Numerous studies investigated survival differences between women and men with MSA [10, 19–21, 23–27, 32–34, 38–40, 42–47, 51, 52]. In 1994, Wenning and colleagues first reported a shorter survival of women with MSA when considering autonomic features—including erectile/ejaculatory dysfunction in men—as initial clinical symptoms, whereas no survival difference was found when marking the disease onset by motor features only [20]. Schrag et al. [33] and O’Sullivan et al. [34] likewise observed a shorter survival of women with MSA; however, both studies also considered male erectile dysfunction as a possible MSA onset feature [34], or did not specify which symptoms qualified for MSA disease onset [33]. A number of more recent studies, which did not account for isolated sexual dysfunction as a MSA onset feature, reported a survival benefit of women with MSA [38, 52], possibly due to an initial motor symptomatic onset and overall less severe autonomic failure compared to men [10]. The majority of the retrieved studies, however, did not observe a sex-related difference in survival [19, 21, 23–27, 32, 39, 40, 42–47].

Based on a shorter time to dependency on walking aids or the wheelchair, an early study of 1998 reported a faster disease progression in women with MSA [25]. Subsequent reports did not observe a sex-related difference in disease progression as indicated by the Unified PD Rating Scale (UPDRS) or UMSARS, or by reaching milestones that correspond to the loss of independent movement [27, 28, 30, 32]. However, two more recent studies, which also applied the UMSARS, again showed a faster disease progression in women [11, 51].

The literature on sex-related differences in the clinical presentation of MSA suggested that compared to men, women more frequently have a motor symptomatic onset and a better response to L-dopa, but overall develop greater motor disability over time and more frequently suffer from pain, depression, anxiety, sarcopenia, frailty, early falls, fractures, worse cognitive performance, and lower health-related quality of life [10, 11, 22, 25, 31, 48, 50, 51, 59]. By contrast, men were more frequently reported to have an autonomic symptomatic onset and to suffer from more widespread and severe autonomic failure, except for urinary urge incontinence and constipation, which were more common in women [10, 12, 20, 34, 36, 37, 41, 51, 55, 60, 61]. Higher supine BP levels as well as greater orthostatic BP falls were both associated with increased mortality in men with MSA [54, 57].

Controversial findings were found in the literature on sex-related differences in excessive daytime sleepiness and REM sleep behavior disorder [53, 56, 61]. The full results of the literature search on sex-related aspects in MSA are provided in ESM Table 2.

### Comparison of the clinical-demographic characteristics of female and male individuals included in the Innsbruck MSA Registry

A total of 56 female (48%) and 60 male (52%) individuals from the Innsbruck MSA Registry fulfilled the inclusion criteria of the present study and were selected for further analysis, while 28 individuals had no follow-up visit and were therefore excluded. No intersex cases were recorded. The women included in the study were younger at the time of disease onset than their male counterparts (56 [50; 63] vs. 62 [53; 66] years; *p* = 0.019). The disease onset was mainly characterized by motor features (i.e., parkinsonian or cerebellar symptoms, in >85% of cases) in both sexes (ESM Table 3).

### Sex-related differences in the clinical presentation at baseline visit

Women also presented at a younger age at the time of the baseline visit (*p* = 0.040). The univariate analysis of clinical characteristics showed that at the baseline visit, women were more likely to suffer from depression (*p* = 0.010) and to be more frequently on CNS-active drugs with BP-lowering side effects (*p* = 0.003). By contrast, men showed a higher frequency of sexual dysfunction (*p* = 0.024) and severe OH (*p* = 0.042). Global disability as indicated by the UMSARS Part IV (more pronounced in women; *p* = 0.067) and cerebellar oculomotor dysfunction (more frequent in men; *p* = 0.093) failed to reach statistical significance, but also qualified as dependent outcome variables for the multivariable regression analysis. The full results of the comparison of clinical characteristics at baseline visit are shown in the left part of Table 1.

**Table 1:** Clinical-demographic characteristics at baseline and last available follow-up of male and female individuals included in the Innsbruck MSA Registry

Clinical-demographic characteristics	Baseline visit			Last follow-up		
	Total MSA cohort (*n* = 116)	Male MSA cohort (*n* = 60)	Female MSA cohort (*n* = 56)	Total MSA cohort (*n* = 116)	Male MSA cohort (*n* = 60)	Female MSA cohort (*n* = 56)
*Sociodemographic data*						
Age (years)	60.9 [55.0; 68.5]	64.0 [55.5; 70.6]*	59.0 [53.2; 65.6]*	64.3 [57.1; 71.3]	66.5 [57.6; 72.9]*	62.4 [56.8; 69.1]*
Disease duration (months)	30.9 [17.2; 48.2]	29.3 [17.4; 45.8]	34.8 [14.2; 53.5]	61.8 [48.7; 83.8]	54.4 [46.3; 70.4]*	68.8 [56.1; 87.9]*
*Motor features and associated treatment*						
Parkinsonism (*n*-BV = 58 M, 55 F; *n*-FU = 57 M, 56 F)	86 (76.1)	46 (79.3)	40 (72.7)	104 (92.0)	50 (87.7)	54 (96.4)
Bradykinesia (*n*-FU = 59 M, 56 F)	90 (77.6)	48 (80.0)	42 (75.0)	107 (93.0)	53 (89.8)	54 (96.4)
Rigidity (*n*-BV = 59 M, 55 F; *n*-FU = 58 M, 56 F)	85 (74.6)	45 (76.3)	40 (72.7)	101 (88.6)	48 (82.8)*	53 (94.6)*
Tremor, any (*n*-BV = 59 M, 56 F)	66 (57.4)	35 (59.3)	31 (55.4)	68 (58.6)	34 (56.7)	34 (60.7)
Postural instability (*n*-BV = 48 M, 49 F; *n*-FU = 52 M, 55 F)	51 (52.6)	23 (47.9)	28 (57.1)	95 (88.8)	43 (82.7)	52 (94.5)
Cerebellar syndrome (*n*-BV = 58 M, 55 F; *n*-FU = 56 M; 53 F)	52 (46.0)	25 (43.1)	27 (49.1)	72 (66.1)	36 (64.3)	36 (67.9)
Gait ataxia (*n*-BV = 60 M, 55 F; *n*-FU = 56 M, 53 F)	57 (49.6)	29 (48.3)	28 (50.9)	74 (67.9)	38 (67.9)	36 (67.9)
Ataxic dysarthria (*n*-BV = 54 M, 52 F; *n*-FU = 47 M, 43 F)	32 (30.2)	14 (25.9)	18 (34.6)	46 (51.1)	21 (44.7)	25 (58.1)
Limb ataxia (*n*-BV = 59 M, 55 F; *n*-FU = 59 M, 55 F)	49 (43.0)	24 (40.7)	25 (45.5)	68 (59.6)	33 (55.9)	35 (63.6)
Cerebellar oculomotor dysfunction	57 (49.1)	34 (56.7)*	23 (41.1)*	88 (75.9)	46 (76.7)	42 (75.0)
Postural abnormalities (*n*-BV = 58 M, 56 F; *n*-FU = 58 M, 56 F)	28 (24.6)	17 (29.3)	11 (19.6)	61 (53.5)	31 (53.4)	30 (53.6)
Pyramidal tract signs (*n*-BV = 54 M, 51 F; *n*-FU = 56 M, 53 F)	58 (55.2)	27 (50.0)	31 (60.8)	61 (56.0)	24 (42.9)*	37 (69.8)*
Dopaminergic treatment	77 (66.4)	42 (70.0)	35 (62.5)	89 (76.7)	43 (71.7)	46 (82.1)
LEDD (mg) (*n*-BV = 41 M, 35 F; *n*-FU = 42 M, 46 F)	600 [300; 975]	583 [300; 947]	600 [300; 1000]	849 [481; 1244]	800 [500; 1169]	938 [400; 1286]
Dopaminergic response (*n*-BV = 38 M, 28 F; *n*-FU = 48 M, 50 F)						
- No or poor	12 (18.2)	5 (13.2)	7 (25.0)	70 (71.4)	39 (81.3)*	31 (62.0)*
- Moderate to good	15 (22.7)	9 (23.7)	6 (21.4)	1 (1.0)	0 (0.0)*	1 (2.0)*
- Uncertain	39 (59.1)	24 (63.2)	15 (53.6)	27 (27.6)	9 (18.7)*	18 (36.0)*
*Rating scales*						
Hoehn and Yahr stage (*n*-BV = 49 M, 45 F; *n*-FU = 56 M, 54 F)	2.3 [2.0; 3.0]	2.0 [2.0; 3.0]	3.0 [2.0; 3.0]	4.0 [3.0; 5.0]	4.0 [2.0; 5.0]	4.0 [4.0; 5.0]
UMSARS Part IV score (*n*-BV = 42 M, 37 F)	2.0 [2.0; 3.0]	2.0 [2.0; 3.0]*	2.0 [2.0; 3.0]*	4.0 [3.0; 4.0]	4.0 [3.0; 4.0]	4.0 [3.0; 5.0]
*Non-motor features & associated treatment*						
Classic OH (*n*-BV = 57 M, 52 F; *n*-FU = 58 M, 53 F)	43 (39.4)	26 (45.6)	17 (32.7)	84 (75.7)	49 (84.5)*	35 (66.0)*
- Severe OH (*n*-BV = 58 M, 51 F; *n*-FU = 56 M, 53 F)	34 (31.2)	23 (39.7)*	11 (21.6)*	67 (61.5)	41 (73.2)*	26 (49.1)*
- History of orthostatic syncope (*n*-BV = 60 M, 53 F; *n*-FU = 59 M, 54 F)	26 (23.0)	17 (28.3)	9 (17.0)	47 (41.6)	30 (50.8)*	17 (31.5)*
- OH treatment, pharmacological and/or non-pharmacological	36 (31.0)	18 (30.0)	18 (32.1)	68 (58.6)	38 (63.3)	30 (53.6)
- OH treatment, pressor agents	26 (22.4)	13 (21.7)	13 (23.2)	50 (43.1)	29 (48.3)	21 (37.5)
- Supine hypertension (*n*-BV = 55 M, 49 F; *n*-FU = 53 M, 54 F)	21 (20.2)	12 (21.8)	9 (18.4)	45 (42.1)	28 (52.8)*	17 (31.5)*
Neurogenic bladder disturbances (*n*-BV = 49 M, 46 F; *n*-FU = 53 M, 49 F)	51 (53.7)	26 (53.1)	25 (54.3)	93 (91.2)	49 (92.5)	44 (89.8)
- Urinary incontinence (*n*-BV = 58 M, 56 F; *n*-FU = 57 M, 55 F)	58 (50.9)	31 (53.4)	27 (48.2)	93 (83.0)	48 (84.2)	45 (81.8)
- Incomplete bladder emptying (*n*-BV = 44 M, 43 F; *n*-FU = 50 M, 42 F)	37 (42.5)	20 (45.5)	17 (39.5)	68 (73.9)	39 (78.0)	29 (69.0)
- Overactive bladder symptoms (*n*-FU = 59 M, 55 F)	78 (67.2)	44 (73.3)	34 (60.7)	109 (95.6)	58 (98.3)	51 (92.7)
- Catheterization (*n*-FU = 59 M, 56 F)	16 (13.8)	9 (15.0)	7 (12.5)	48 (41.7)	29 (49.2)*	19 (33.9)*
Sexual dysfunction (*n*-BV = 30 M, 6 F; *n*-FU = 43 M, 17 F)	31 (86.1)	28 (93.3)*	3 (50.0)*	57 (95.0)	43 (100)*	14 (82.4)*
Constipation (*n*-BV = 47 M, 46 F; *n*-FU = 50 M, 49 F)	43 (46.2)	22 (46.8)	21 (45.7)	75 (75.8)	39 (78.0)	36 (73.5)
Stridor (*n*-BV = 16 M, 22 F; *n*-FU = 29 M, 25 F)	8 (21.1)	2 (12.5)	6 (27.3)	29 (53.7)	15 (51.7)	14 (56.0)
Inspiratory sighs (*n*-BV = 14 M, 14 F; *n*-FU = 21 M, 21 F)	8 (28.6)	4 (28.6)	4 (28.6)	25 (59.5)	12 (57.1)	13 (61.9)
Speaking/acting out dreams (*n*-BV = 44 M, 37 F; *n*-FU = 55 M, 51 F)	51 (63.0)	28 (63.6)	23 (62.2)	83 (78.3)	41 (74.5)	42 (82.4)
Depression (*n*-BV = 57 M, 48 F; *n*-FU = 58 M, 48 F)	49 (46.7)	20 (35.1)*	29 (60.4)*	63 (59.4)	24 (41.4)*	39 (81.3)*
Intake of CNS-active drugs with BP lowering side effects	60 (51.7)	23 (38.3)*	37 (66.1)*	77 (66.4)	30 (50.0)*	47 (83.9)*
*Comorbidities*						
Cardiovascular disease	41 (35.3)	25 (41.7)	16 (28.6)	49 (42.2)	31 (51.7)*	18 (32.1)*
Antihypertensive medication intake	36 (31.0)	21 (35.0)	15 (26.8)	39 (33.6)	29 (48.3)*	10 (17.9)*
Diabetes mellitus (*n*-BV = 59 M, 56 F; *n*-FU = 59 M, 56 F)	11 (9.6)	7 (11.9)	4 (7.1)	12 (10.4)	8 (13.6)	4 (7.1)

Qualitative variables are presented as the absolute frequency (percentage) and quantitative variables as the median [25th; 75th percentile]

*BP* blood pressure, *CNS* central nervous system, *F* female, *LEDD* L-dopa equivalent daily dosage, *M* male, *MSA* multiple system atrophy, *n* number, *n-BV* number at baseline visit, *n-FU* number at last follow-up, *OH* orthostatic hypotension, *UMSARS* Unified MSA Rating Scale

*Significant at *p* < 0.1 in the comparison between men and women

Multivariable regression analysis showed that female sex was independently associated with depression at baseline. By contrast, male sex was associated with the presence of cerebellar oculomotor dysfunction (this, in turn, was associated with a cerebellar symptomatic onset and a longer disease duration to baseline visit), severe OH (which was negatively associated with the intake of antihypertensive medications), and sexual dysfunction. Table 2 summarizes the clinical characteristics that showed an association with female or male sex in the multivariable analysis, while also indicating other associated covariates. The multivariable analysis did not reveal an association of sex with the baseline level of global disability (i.e., UMSARS Part IV score) or the intake of CNS-active drugs with BP-lowering side effects anymore (ESM Table 4).

**Table 2:** Clinical characteristics of Innsbruck MSA individuals that showed an association with female or male sex in the multivariable analysis

Outcome variable (i.e., clinical characteristic showing an association with female or male sex)	Covariate sex			Other covariates in the multivariable models
	M/F	Odds ratio (95% CI)	*p*	
*At baseline visit*				
Cerebellar oculomotor dysfunction (yes vs. no)	M	2.7 (1.2–6.5)	0.021*	Initial clinical feature: cerebellar symptoms^b^. At baseline visit: age; disease duration^b^
Severe OH (yes vs. no)	M	5.5 (1.4–21.9)	0.016*	At baseline visit: age; disease duration; LEDD; intake of CNS-active drugs with BP lowering side effects; antihypertensive medication intake^c^
Sexual dysfunction (yes vs. no)	M	14 (1.6–120.1)^a^	0.024*	At baseline visit: age; disease duration; depression; comorbidities (cardiovascular disease; diabetes mellitus)
Depression (yes vs. no)	F	4.7 (1.5–14.1)	0.007*	At baseline visit: age; disease duration; UMSARS Part IV score; comorbidities (cardiovascular disease; diabetes mellitus)
*At last available follow-up*				
Pyramidal tract signs (yes vs. no)	F	2.7 (1.2–6.2)	0.019*	At last follow-up: age; disease duration; comorbidities (diabetes mellitus)
Classic OH (yes vs. no)	M	7.5 (1.5–37.8)	0.014*	At last follow-up: age; disease duration; LEDD; intake of CNS-active drugs with BP-lowering side effects; antihypertensive medication intake^c^
Severe OH (yes vs. no)	M	11.1 (2.5–50.2)	0.002*	At last follow-up: age; disease duration; LEDD; intake of CNS-active drugs with BP-lowering side effects; antihypertensive medication intake
Supine hypertension (yes vs. no)	M	3.0 (1.2–7.6)	0.020*	At last follow-up: age; disease duration; OH treatment (pressor agents); antihypertensive medication intake
Depression (yes vs. no)	F	6.2 (2.3–16.4)	<0.001*	At last follow-up: age; disease duration; UMSARS Part IV score; comorbidities (cardiovascular disease; diabetes mellitus)
Intake of CNS-active drugs with BP-lowering side effects (yes vs. no)	F	4.1 (1.2–14.4)	0.029*	At last follow-up: age; disease duration^c^; UMSARS Part IV score^b^; depression^b^; speaking/acting out dreams
Antihypertensive medication intake (yes vs. no)	M	8.7 (2.4–32.2)	0.001*	At last follow-up: age; disease duration; severe OH; supine hypertension; comorbidities (cardiovascular disease^b^)
*Change from baseline to last available follow-up*				
Antihypertensive medication intake (start yes vs. no)	M	12.4 (1.5–99.1)^a^	0.018*	Age at last follow-up; follow-up time New onset from baseline to follow-up of: severe OH^b^; supine hypertension^c^; comorbidities (cardiovascular disease^b^)

*BP* blood pressure, *CI* confidence interval, *F* female, *LEDD* L-dopa equivalent daily dosage, *M* male, *MSA* multiple system atrophy, *OH* orthostatic hypotension, *UMSARS* Unified MSA Rating Scale

*Significant at *p* < 0.05

^a^Presentation of bivariate odds ratio

^b^Other covariates included in the multivariable analysis and showing a significant positive association with the outcome variable (underlined)

^c^Other covariates included in the multivariable analysis and showing a significant negative association with the outcome variable (underlined)

### Sex-related differences in the clinical presentation at last available follow-up

At last available follow-up, 99 (85%) individuals fulfilled the criteria for probable MSA and 73 were classified as MSA-P (63%), without any sex-related differences observed in the level of MSA diagnostic certainty (45 [80%] women were diagnosed with probable MSA vs. 54 [90%] men; *p* = 0.142) or predominant MSA subtype (35 [63%] women with MSA-P vs. 38 [63%] men; *p* = 0.926).

Compared to men, women showed a longer disease duration up to the last available follow-up (*p* = 0.005). Women also had a higher frequency of depression (*p* < 0.001), were more frequently on CNS-active drugs with BP-lowering side effects (*p* < 0.001), and were more likely to show pyramidal signs at last available follow-up (*p* = 0.005). By contrast, men showed a higher frequency of classic (*p* = 0.024) and severe OH (*p* = 0.010), orthostatic syncope (*p* = 0.037), supine hypertension (*p* = 0.025), and sexual dysfunction (*p* = 0.020). Men also had a higher prevalence of cardiovascular comorbidities (*p* = 0.033) and a more frequent intake of antihypertensive medication (*p* < 0.001). Other characteristics that qualified for the multivariable regression analysis comprised rigidity (more frequent in women; *p* = 0.075), dopaminergic response (worse in men; *p* = 0.055), and catheterization (more frequent in men; *p* = 0.098). Full results of the comparison of clinical characteristics at last available follow-up are shown in the right part of Table 1.

Multivariable regression analysis showed that at last follow-up, female sex was independently associated with pyramidal signs and depression (Table 2). In contrast to the baseline visit, at last follow-up, female sex showed an association with the intake of CNS-active drugs with BP-lowering side effects (this, in turn, was associated with a higher UMSARS Part IV score, depression, and a shorter disease duration to last follow-up). Male sex was associated with classic OH (which also showed a negative association with the intake of antihypertensive medication), severe OH, and supine hypertension. In addition, male sex showed an association with the intake of antihypertensive medication (which was also associated with the presence of cardiovascular comorbidities). We did not observe an association between sex and rigidity, response to dopaminergic treatment, history of orthostatic syncope, catheterization, or cardiovascular comorbidities at last available follow-up (ESM Table 4).

### Sex-related differences in the baseline to last follow-up changes in clinical presentation

The available follow-up time remained comparable between sexes (Table 3). Over the observational period, women had a higher increase of L-dopa equivalent daily dosage, whereas men showed a more frequent start of antihypertensive medication. Other characteristics that qualified for the multivariable regression analysis included development of parkinsonism or bradykinesia (in individuals with the MSA-C phenotype), rigidity, and postural abnormalities (all more frequent in women), as well as new onset of supine hypertension and worsening of dopaminergic response (both more frequent in men).

**Table 3:** Baseline to last available follow-up change of clinical characteristics and follow-up time in male versus female individuals included in the Innsbruck MSA Registry

Clinical presentation	Total MSA cohort (*n* = 116)	Male MSA cohort (*n* = 60)	Female MSA cohort (*n* = 56)	*p* (M vs. F)
*Sociodemographic data*				
Follow-up time (months)	27.0 [14.3; 41.8]	25.5 [11.4; 39.1]	29.8 [14.9; 47.2]	0.193
*Motor features & associated treatment*				
New onset of parkinsonism (*n* = 56 M; 55 F)	19 (17.1)	6 (10.7)	13 (23.6)	0.071*
New onset of bradykinesia (*n* = 59 M; 56 F)	18 (15.7)	6 (10.2)	12 (21.4)	0.097*
New onset of rigidity (*n* = 57 M; 55 F)	19 (17.0)	6 (10.5)	13 (23.6)	0.065*
New onset of tremor, any (*n* = 59 M; 56 F)	19 (16.5)	8 (13.6)	11 (19.6)	0.380
New onset of postural instability (*n* = 46 M; 48 F)	32 (34.0)	15 (32.6)	17 (35.4)	0.774
New onset of cerebellar syndrome (*n* = 55 M; 52 F)	19 (17.8)	11 (20.0)	8 (15.4)	0.532
New onset of gait ataxia (*n* = 56 M; 52 F)	17 (15.7)	10 (17.9)	7 (13.5)	0.531
New onset of ataxic dysarthria (*n* = 47 M; 42 F)	14 (15.7)	8 (17.0)	6 (14.3)	0.723
New onset of limb ataxia (*n* = 58 M; 54 F)	24 (21.4)	12 (20.7)	12 (22.2)	0.843
New onset of cerebellar oculomotor dysfunction	37 (31.9)	16 (26.7)	21 (37.5)	0.211
New onset of postural abnormalities (*n* = 56 M; 56 F)	32 (28.6)	12 (21.4)	20 (35.7)	0.094*
New onset of pyramidal tract signs (*n* = 51 M; 49 F)	18 (18.0)	7 (13.7)	11 (22.4)	0.256
Start of dopaminergic treatment	17 (14.7)	6 (10.0)	11 (19.6)	0.142
- Adjustment of LEDD (mg) (*n* = 46 M; 46 F)	290.0 [-33.0; 490.0]	195.0 [-92.5; 396.3]	337.5 [1.8; 587.5]	0.013*
- Worsening of dopaminergic response (*n* = 38 M; 28 F)	45 (68.2)	29 (76.3)	16 (57.1)	0.098*
*Rating scales*				
Increase of Hoehn and Yahr, overall (*n* = 56 M; 54 F)	89 (80.9)	42 (75.0)	47 (87.0)	0.108
Increase of Hoehn and Yahr stage (*n* = 56 M; 54 F)	2.0 [1.0; 3.0]	1.0 [0.0; 3.0]	2.0 [1.0; 3.0]	0.120
- per year of follow-up (*n* = 56 M; 54 F)	0.6 [0.3; 1.0]	0.6 [0.0; 0.9]	0.6 [0.4; 1.1]	0.849
Increase of UMSARS Part IV, overall (*n* = 42 M; 37 F)	61 (77.2)	31 (73.8)	30 (81.1)	0.442
Increase of UMSARS Part IV score (*n* = 42 M; 37 F)	1.0 [1.0; 2.0]	1.0 [0.0; 2.0]	2.0 [1.0; 2.0]	0.327
- per year of follow-up (*n* = 42 M; 37 F)	0.6 [0.3; 1.0]	0.6 [0.0; 1.1]	0.7 [0.4; 0.9]	0.916
*Non-motor features & associated treatment*				
New onset of classic OH (*n* = 56 M; 51 F)	38 (35.5)	21 (37.5)	17 (33.3)	0.653
- New onset of severe OH (*n* = 55 M; 49 F)	29 (27.9)	17 (30.9)	12 (24.5)	0.466
- New onset of history of orthostatic syncope (*n* = 59 M; 52 F)	20 (18.0)	13 (22.0)	7 (13.5)	0.241
- Start of OH treatment, pharmacological and/or non-pharmacological	36 (31.0)	20 (33.3)	16 (28.6)	0.580
- Start of OH treatment, pressor agents	29 (25.0)	18 (30.0)	11 (19.6)	0.198
- New onset of supine hypertension (*n* = 49 M; 48 F)	19 (19.6)	13 (26.5)	6 (12.5)	0.082*
New onset of neurogenic bladder disturbances (*n* = 46 M; 43 F)	29 (32.6)	16 (34.8)	13 (30.2)	0.647
- New onset of urinary incontinence (*n* = 56 M; 55 F)	36 (32.4)	18 (32.1)	18 (32.7)	0.948
- New onset of incomplete bladder emptying (*n* = 40 M; 38 F)	21 (26.9)	10 (25.0)	11 (28.9)	0.694
- New onset of overactive bladder symptoms (*n* = 59 M; 55 F)	32 (28.1)	15 (25.4)	17 (30.9)	0.515
- Start of catheterization (*n* = 59 M; 56 F)	34 (29.6)	21 (35.6)	13 (23.2)	0.146
New onset of sexual dysfunction (*n* = 30 M; 6 F)	2 (5.6)	2 (6.7)	0 (0)	1.000
New onset of constipation (*n* = 42 M; 41 F)	23 (27.7)	13 (31.0)	10 (24.4)	0.504
New onset of stridor (*n* = 15 M; 18 F)	2 (6.1)	1 (6.7)	1 (5.6)	1.000
New onset of inspiratory sighs (*n* = 14 M; 13 F)	2 (7.4)	1 (7.1)	1 (7.7)	1.000
New onset of speaking/acting out dreams (*n* = 43 M; 37 F)	11 (13.8)	4 (9.3)	7 (18.9)	0.330
New onset of depression (*n* = 56 M; 43 F)	15 (15.2)	7 (12.5)	8 (18.6)	0.401
Start of CNS-active drugs with BP lowering side effects	23 (19.8)	11 (18.3)	12 (21.4)	0.676
*Comorbidities*				
New onset of cardiovascular disease	9 (7.8)	6 (10.0)	3 (5.4)	0.493
Start of antihypertensive medication intake	12 (10.3)	11 (18.3)	1 (1.8)	0.004*
New onset of diabetes mellitus (*n* = 59 M; 56 F)	1 (0.9)	1 (1.7)	0 (0)	1.000

Qualitative variables are presented as the absolute frequency (percentage) and quantitative variables as the median [25th; 75th percentile]

*BP* blood pressure, *CNS* central nervous system, *F* female, *LEDD* L-dopa daily equivalent dosage, *M* male, *MSA* multiple system atrophy, *n* number, *OH* orthostatic hypotension, *UMSARS* Unified MSA Rating Scale

*Significant at *p* < 0.1 in the comparison between men and women

In the multivariable model, male sex was associated with the introduction of antihypertensive medications over the observational period (Table 2), while we did not observe an association of sex with newly developed parkinsonism, bradykinesia, rigidity, postural abnormalities, supine hypertension, a L-dopa equivalent daily dosage increase of more than 300 mg/day, or worsening of dopaminergic response (ESM Table 4).

## Discussion

Although sex-related differences generally remain understudied in MSA, we found a steadily increasing number of publications assessing—at least in subgroup analyses—the influence of sex on the clinical presentation and natural history of MSA. Such mounting research interest is probably due to the overall growing awareness of the importance of considering sex-specific aspects in healthcare provision.

The most frequently investigated sex-related aspect in the MSA literature was survival, with the majority of studies concluding on a comparable survival outcome between sexes [19, 20, 23–27, 32, 39, 40, 42–47]. In these studies, disease onset was mainly defined with the beginning of motor features only. Earlier studies reported on a male survival benefit, but also considered sexual dysfunction for defining MSA disease onset [20, 34]. While erectile dysfunction is a common and well-recognized early feature in men with MSA, female sexual dysfunction remains largely underestimated and only rarely addressed, despite its high prevalence [9]. These earlier reports of a male survival benefit were therefore probably due to differential recording of sexual dysfunction in women and men and likely affected by a lead-time bias [20, 24, 34].

The literature on sex-related differences in the clinical presentation of MSA primarily consisted of studies conducted following the publication of the second consensus MSA criteria [14]. Altogether, these studies indicated that women suffer from a greater motor disability and higher neuropsychiatric burden, while possibly experiencing a slight survival benefit due to initial motor onset and overall less severe autonomic failure compared to men [10–12, 38, 41, 51, 52, 54, 55, 57, 60, 61]. A motor onset in MSA may positively impact women’s survival by prompting an earlier referral, diagnosis, and thus optimized care [10]. By contrast, early and severe autonomic failure has been  associated with increased cardiovascular morbidity and mortality both in MSA and in the general population [27, 34, 42, 43, 63–65], and may explain the shorter survival of male MSA individuals observed in some recent studies [10, 54].

In the present retrospective study, we focused on sex-related differences in the clinical presentation of MSA and—for the first time—its evolution over time. We found that:

1. Cardiovascular autonomic failure occurred more frequently and was more severe in men. As early as at baseline, men suffered more frequently from severe OH, while at follow-up, both severe OH and supine hypertension were independently associated with male sex.
2. Male sex was associated with a higher use of antihypertensive medications throughout the observation period.
3. In contrast to men, depression was more frequent in women and independently associated with female sex both at baseline and last available follow-up.
4. Female sex was associated with the intake of CNS-active drugs with BP-lowering side effects at last follow-up.

Population-based studies generally indicate a similar prevalence of OH among aging women and men [66–68]. However, in individuals aged > 75 years, a higher OH frequency has been reported in men [69]. OH has also been observed more frequently in men with PD [70], especially in male individuals of taller stature [71]. A greater height is generally associated with lower BP in healthy elderly individuals, possibly reflecting underlying hydrostatic mechanisms [72], and a taller stature may likewise contribute to an increased risk of developing clinically relevant orthostatic BP falls in men [73]. The influence of sex on autonomic BP control in aging individuals also needs to be considered [74]. Vascular transduction, i.e., more vasoconstriction/a greater increase in BP for a given sympathetic burst, has been shown to increase with age in women (in part possibly due to the loss of estrogen), while decreasing in men [74]. This increase in sympathetic activity and adrenergic vasoconstrictor responsiveness may partially compensate for the changes arising from MSA neurodegeneration at a preganglionic level. Altogether, sex-specific morphological and physiological differences in cardiovascular autonomic BP control may explain the predisposition of men with MSA to develop earlier and more severe cardiovascular autonomic failure.

We also found that both classic and severe OH were negatively associated with an antihypertensive medication intake. Antihypertensive drugs are among the most frequent factors exacerbating OH, and reducing the dosage or suspending their administration is an established initial step of OH management [75]. This negative association therefore likely reflects the implementation of a stepwise OH treatment approach in a tertiary referral center [75, 76].

Compared to the general aging population, in which women and men are equally likely to use antihypertensive medication for cardiovascular disease [77], the here observed association of an antihypertensive medication intake with male sex over time and at last follow-up may be driven by a higher frequency of supine hypertension in male MSA subjects.

Although men had a higher prevalence of cardiovascular comorbidities at last follow-up, the multivariable analysis only showed an association between older age and the occurrence of cardiovascular comorbidities. For individuals aged 60 to 79 years, data from the *2023 Heart Disease and Stroke Statistics Update* [78] in fact shows that age-adjusted frequency rates of cardiovascular comorbidities remain similar between sexes. The higher frequency of cardiovascular comorbidities in our male MSA cohort might therefore be due to the higher age at disease onset and subsequently also the borderline higher age at last follow-up of men. Despite previous studies showing a link between cardiovascular autonomic failure and worse cardio- and cerebrovascular outcomes in affected individuals [63, 79, 80], we did not observe an association between cardiovascular comorbidities and severe OH or supine hypertension in our MSA cohort. This finding may result from the limited follow-up time of the present study, since end-organ damage may cumulate over a long time before it becomes clinically manifest.

Meta-analyses and population-based studies suggest that women are diagnosed with depression about twice as often as men [81–83]. Frequency rates of depression in MSA are significantly higher when compared to age- and sex-matched healthy controls [84], and a preponderance of depression in women with MSA has been reported in earlier studies [11, 48]. The here observed higher frequency of depression in women and its independent association with female sex thus likely reflect a sex-related female background susceptibility to developing mood disorders, amplified by the burden of a relentlessly progressing disease. In addition, gender-related differences in receiving home-care versus undergoing early institutionalization and a higher likelihood of recognizing depression in women may possibly contribute to this observation [84].

Anti-depressants represented a considerable proportion of the CNS-active drugs with BP-lowering side effects observed in the present study and were associated with both depression and female sex at last available follow-up. This observation may indicate that women are more prone to seek mental health care and/or have a more favorable response to antidepressant therapy [83, 85–88].

Compared to previous studies [10, 12, 37, 55], we did not observe an association between sex and urinary incontinence or catheterization. In the general population, perineal laxity and multiparity are well-known factors that contribute to urinary incontinence in women, while prostate hypertrophy may cause urinary retention in men. These sex-specific anatomical differences are also likely to contribute to the occurrence and severity of urological disturbances in individuals with MSA, emphasizing the need for further research in this area.

This study has a number of limitations. During the scoping phase, we carefully assessed the feasibility of performing a systematic review, but given the high heterogeneity of the data and possible overlap across the available studies, we ultimately sought to provide a narrative overview of the topic. Due to the monocentric and retrospective assessment of the MSA cohort, potential selection, observer, recall, and documentation biases are also to be considered. However, all assessments were performed by staff members with long-standing expertise in the field of atypical parkinsonian disorders, and often repeated multiple times over the follow-up period, thereby securing accurate and comprehensive information for most of the reported clinical characteristics.

Documentation inhomogeneity particularly affected non-motor symptoms, such as stridor, inspiratory sighs, and sexual dysfunction. The latter was by far better documented in male than female MSA individuals, probably reflecting a sociodynamic, gender-related difference in history-taking. The retrospective Innsbruck MSA Registry also did not include any transgender cases nor were closer gender-related aspects documented, thereby preventing an assessment of their possible influence on medical decision-making in MSA. Moreover, cardiovascular and urological autonomic testing was not performed exclusively in our center. Whenever available, the respective findings were taken into account for a qualitative analysis, but the lack of biosignal homogeneity hindered a quantitative analysis of autonomic function indices. Likewise, full UMSARS scores were only available in a subset of patients and could therefore not be included in the data analysis.

In our MSA cohort, men had a significantly higher age at disease onset. To counteract this potential source of bias, we included the respective age as well as the disease duration or available follow-up time in the multivariable analyses. Interestingly, in PD, women tend to have a later disease onset than men, possibly due to neuroprotective effects of female sex hormones, especially estrogen [6, 89, 90]. While it remains unclear how sex hormones may influence α-synuclein pathology and studies in MSA models are lacking, our findings suggest that female sex hormones may lack a neuroprotective effect against MSA-specific mechanisms of disease.

Since sex has been recently shown to have no significant effect on survival in a larger sample of Innsbruck MSA patients [91], we did not perform another survival analysis. Finally, given the explorative, rather than hypothesis-driven, nature of the study, we did not correct for multiple testing, but performed multivariable analyses to evaluate the influence of putative demographic and clinical confounders on sex-related differences in MSA core clinical features and treatment choices observed at the univariate analysis. The converging evidence from both clinical and neuropathological studies ultimately substantiates the sex-related differences observed in our study.

## Conclusion

The available literature and findings from our retrospective series indicate that sex-related differences do exist in the clinical presentation of MSA and its symptoms' evolution over time. While early and severe cardiovascular autonomic failure was more frequent in our male MSA cohort, our female MSA cohort suffered from a higher burden of psychiatric comorbidities. These differences in the symptomatic profile likely reflect sex-specific morphological and biological background susceptibilities to such disorders, amplified by the MSA neurodegenerative changes and disease burden.

Awareness for sex-related differences in the MSA disease journey holds promise for improving therapeutic decision-making to a highly individualized level. Future research efforts are equally called to integrate sex-related outcome measures and assess the prognostic relevance of sex-related differences in MSA clinical presentation. Ultimately, a better understanding of MSA sex-related aspects will guide the development of treatment strategies and prevention of complications that meet the different needs of women and men living with MSA.

### Supplementary Information

Below is the link to the electronic supplementary material.

Supplementary file1 (PDF 724 KB)

## Data Availability

The first and last author take responsibility for the integrity of the data presented herein. The data supporting the findings of this study are available upon reasonable request from any of the qualified investigators.
