# Supplementary material for: Sex-related differences in the clinical presentation of multiple system atrophy
Source: Clin Auton Res. 2024 Apr 17;34(2):253–68. doi: 10.1007/s10286-024-01028-1 (PMC11127878; doi:10.1007/s10286-024-01028-1)
Supplement: Supplementary file 1 — Supplementary file1 (PDF 724 KB) [file 10286_2024_1028_MOESM1_ESM.pdf]

Supplementary Material

Sex-related differences in the clinical presentation of multiple system atrophy

Fabian Leys MD PhD[1], Sabine Eschlböck MD[1,2], Nicole Campese MD[1], Philipp Mahlknecht MD PhD[1], Marina Peball MD PhD[1], Georg Goebel PhD[3], Victoria Sidoroff MD[1], Florian Krismer MD PhD[1], Roberta Granata MD[1], Stefan Kiechl MD[1], Werner Poewe MD[1], Klaus Seppi MD[1,4], Gregor K. Wenning MD PhD MSc[1] and Alessandra Fanciulli MD PhD[1]

[1]*Department of Neurology, Medical University of Innsbruck, Innsbruck, Austria*
[2]*Department of Neurology, Hochzirl-Natters Hospital, Zirl, Austria*
[3]*Institute of Medical Statistics and Informatics, Medical University of Innsbruck, Innsbruck, Austria*
[4]*Department of Neurology, Provincial Hospital of Kufstein, Kufstein, Austria*

Supplementary Figure 1 Flow chart of the literature search....2

Supplementary Table 1 Definition of the investigated clinical-demographic characteristics 3

Supplementary Figure 2 Flow chart and analytic approach of the statistical analysis....9

Supplementary Table 2 Literature findings with sex-related information in MSA....10

Supplementary Table 3 Disease onset characteristics of male and female individuals included in the Innsbruck MSA Registry 21

Supplementary Table 4 Clinical characteristics of Innsbruck MSA individuals showing no association with female or male sex in the multivariable analysis....22

STrengthening the **R**eporting of **OB**servational studies in **E**pidemiology (STROBE) **Statement** 24

Corresponding author:

Assist.-Prof. Alessandra Fanciulli, MD PhD – Department of Neurology, Medical University of Innsbruck

✉ alessandra.fanciulli@i-med.ac.at

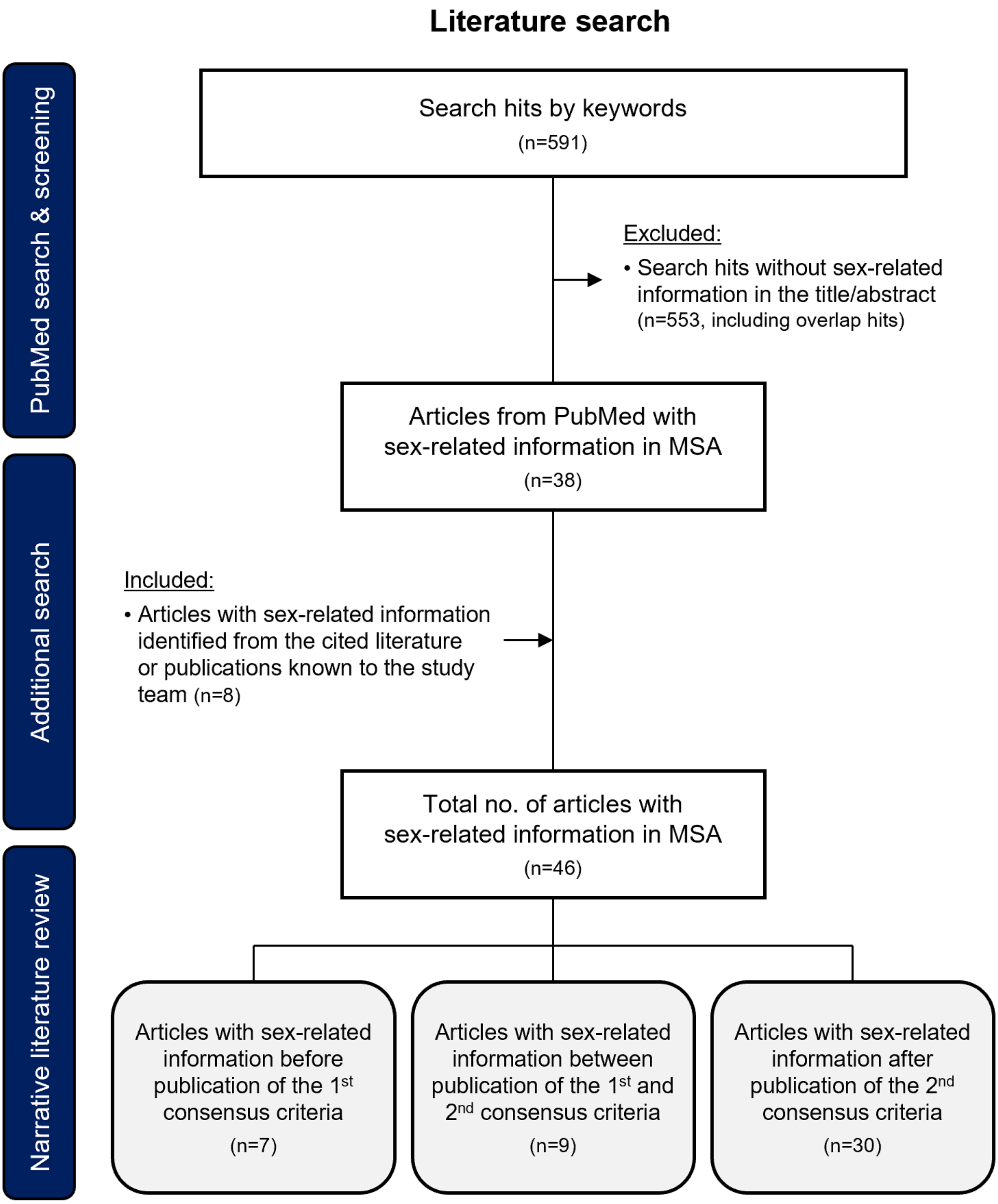

Supplementary Fig. 1 Flow chart of the literature search

Supplementary Table 1 Definition of the investigated clinical-demographic characteristics

Variable	Definition
Sociodemographic data	
Sex	Documented sex: female or male.
Age at onset	Age at the time of the initial clinical feature(s) in years.
Age at baseline visit	Age at baseline visit in years.
Disease duration at baseline visit	Time from age at onset to the baseline visit in months.
Age at last follow-up	Age at last follow-up in years.
Disease duration at last follow-up	Time from age at onset to the last follow-up in months.
Follow-up time	Time from the baseline visit to the last follow-up in months.
Initial clinical feature(s)	
Motor	Disease onset characterized by motor features (i.e., parkinsonian or cerebellar symptoms).
Autonomic	Disease onset characterized by autonomic features (i.e., orthostatic intolerance or bladder disturbances).
Motor & autonomic	Disease onset characterized by a combination of motor and autonomic features (if both were reported to have occurred simultaneously) [1].

Motor features & associated treatment	
Parkinsonism	Documented in the neurological examination: bradykinesia plus at least one out of muscular rigidity, tremor, or postural instability not caused by primary visual, vestibular, cerebellar, or proprioceptive dysfunction [2].
Bradykinesia	Documented in the neurological examination: slowness of movements with progressive reduction in speed and amplitude of repetitive actions [3].
Rigidity	Documented in the neurological examination: muscular rigidity, i.e., documented “cogwheel” sign.
Tremor, any	Documented in the neurological examination: either resting, postural/action, or jerky myoclonic tremor.
Postural instability	Documented in the neurological examination: if the patient needed more than three steps to compensate the pull-test or was about to fall if not caught by the examiner [4].<br>In case that testing for postural instability was not documented or assessable, e.g., due to advanced motor impairment or a wheelchair-bound stage, “yes” was applied to this variable whenever history and clinical global impression strongly indicated its presence.
Cerebellar syndrome	Documented in the neurological examination: gait ataxia with at least one of ataxic dysarthria, limb ataxia, or cerebellar oculomotor dysfunction (i.e., saccadic eye movements or pathologic nystagmus) [2].<br>In case that gait ataxia was not documented or assessable, e.g., due to a wheelchair-bound stage, a cerebellar syndrome was considered if other cerebellar signs were unequivocally present.
Gait ataxia	Documented in the neurological examination: ataxic, broad-based gait.
Ataxic dysarthria	Documented in the neurological examination: presence of scanning dysarthria.<br>This variable was considered as missing whenever the type of dysarthria was not clearly documented or assessable.
Limb ataxia	Documented in the neurological examination: ataxia of the upper or lower extremities as documented at the finger-nose, finger-finger or heel-shin test.
Cerebellar oculomotor dysfunction	Documented in the neurological examination: saccadic eye movements (i.e., broken-up smooth pursuit, saccadic hypermetria) and/or pathological nystagmus (i.e., gaze-evoked nystagmus of more or less than 45 degrees) [4].
Postural abnormalities	Documented in the neurological examination: disproportionate antecollis, camptocormia, or Pisa syndrome.

Pyramidal tract signs	Documented in the neurological examination: Babinski sign, hyperreflexia, or spasticity.
Dopaminergic treatment	Documented dopaminergic treatment, i.e., dopamine agonists, MAO-B inhibitors, L-dopa, with or without COMT-inhibitors, and amantadine.
L-dopa equivalent daily dosage	The L-dopa equivalent daily dosage was calculated with https://www.parkinsonsmeasurement.org/toolBox/levodopaEquivalentDose.htm based on documented dopaminergic treatment [5].<br>For the multivariable binary regression analysis of the change from baseline to last available follow-up, we parted the adjustment of LEDD in < / ≥ 300 mg/day (i.e., reduction or maximum increase of <299 mg/day in LEDD versus a clinically meaningful LEDD increase of ≥300 mg/day).
Dopaminergic response	Dopaminergic responsiveness was defined as previously described by Fanciulli *et al*. [6], i.e.:<br>no or poor response: if both the patient and the treating clinician agreed on the lack of or only poor benefit from dopaminergic treatment;<br>moderate to good response: if both the patient and the treating clinician reported (or observed) benefit from dopaminergic treatment;<br>uncertain response: if there was disagreement between the patient's report and the clinician's observation or if the changes observed were insufficient to draw conclusions on efficacy (e.g., due to low total daily dopaminergic dosage, insufficient follow-up time, or reduced compliance because of side effects).<br>For the multivariable binary regression analysis at available last follow-up, we divided the dopaminergic response in "moderate to good, or uncertain response" versus "no or poor response".
Rating scales	
Hoehn & Yahr stage	If available, the Hoehn & Yahr stage was taken over from the medical records or, otherwise, post-hoc assessed according to the modified Hoehn & Yahr scale [7] based on the clinical neurological examination and medical history.<br>Presence of at least one feature suggesting parkinsonism [2, 3] was required to assess the Hoehn & Yahr stage.
Unified MSA Rating Scale Part IV score	If available, the Unified MSA Rating Scale Part IV [4] score at was taken over from the medical records or, otherwise, post-hoc assessed based on the clinical neurological examination and medical history.<br>For the multivariable binary regression analysis at baseline visit, we parted the Unified MSA Rating Scale Part IV score in ≤ / > 2 (i.e., ≤2=fully independent or requiring some help with core activities versus >2=more to totally dependent in core activities).

Non-motor features & associated treatment	
Classic orthostatic hypotension	Documented diagnosis of orthostatic hypotension or available orthostatic challenge test showing blood pressure falls of ≥20 mmHg in systolic or ≥10 mmHg in diastolic values within three minutes of active standing or passive head-up tilt [8].
Severe orthostatic hypotension	Documented diagnosis of orthostatic hypotension [8] with history of recurrent syncope, available orthostatic challenge test showing either a blood pressure fall of ≥30 mmHg in systolic or ≥15 mmHg in diastolic values within three minutes of active standing or passive head-up tilt, or orthostatic challenge test having to be interrupted because of presyncope.
History of orthostatic syncope	Documented history of syncope due to orthostatic hypotension.
Orthostatic hypotension treatment, pharmacological and/or non-pharmacological	Documented pharmacologic and/or non-pharmacologic treatment for orthostatic hypotension [9].
Orthostatic hypotension treatment, pressor agents	Documented pharmacologic treatment for orthostatic hypotension, i.e., intake of pressor agents (i.e., midodrine, fludrocortisone, droxidopa, ethylephrine).
Supine hypertension	In case of orthostatic hypotension: documented diagnosis of supine hypertension or supine blood pressure readings fulfilling consensus diagnostic criteria for supine hypertension [10].
Neurogenic bladder disturbances	Documented urinary urge incontinence or incomplete bladder emptying without relation to possible secondary/non-neurogenic causes.
Urinary incontinence	Documented urinary incontinence.
Incomplete bladder emptying	Documented bladder voiding disorder or measured post-void residual volumes of ≥100 ml.
Overactive bladder symptoms	Documented urge incontinence, urinary urgency, or increased urinary frequency.

Catheterization	Documented need of intermittent or suprapubic catheterization.
Sexual dysfunction	Documented erectile dysfunction in male individuals or desire, arousal, lubrication, genital sensitivity disturbances in female ones.
Constipation	History of less than three bowel movements per week or laxatives intake.
Stridor	Documented inspiratory stridor, including polysomnography-confirmed inspiratory stridor.
Inspiratory sighs	Documented inspiratory sighs.
Speaking/acting out dreams	Documented history of speaking / acting out dreams suggesting REM sleep behavior disorder.
Depression	Documented diagnosis of depression, significantly depressed mood, or anti-depressants intake (low dose antidepressants taken at bedtime for hypnotic purposes, e.g., low-dose trazodone or mirtazapine, did not account for a depression diagnosis).
Intake of CNS-active drugs with blood pressure lowering side effects	Beyond dopaminergic medication, documented intake of CNS-active drugs with blood pressure lowering side effects as defined by Rivasi *et al.* (i.e., anti-depressants, benzodiazepines, anti-psychotics, opioids, and trazodone) [11].
Comorbidities	
Cardiovascular disease	Documented diagnosis of any cardio- and/or cerebrovascular disease, including arterial hypertension [12].
Antihypertensive medication intake	Documented intake of antihypertensive medication, i.e., diuretics, nitrates, (combined) α- and/or β-blockers, calcium channel blockers, central α-2 agonists, ACE inhibitors, and angiotensin receptor type II blockers [11, 13].
Diabetes mellitus	Documented diagnosis of diabetes mellitus.

When parts of the neurological or other examinations, e.g., the pull or active orthostatic challenge test, were not repeated at last follow-up due to, e.g., a wheelchair-bound stage, the last observation carried forward principle was used for all features associated with the neurodegenerative nature of the disease, i.e., those that once established, such as postural instability, are unlikely to recover anymore.
LEDD, L-dopa equivalent daily dosage; MSA, multiple system atrophy.
In part reproduced with adaptations with the permission of Leys *et al.*[14] (https://creativecommons.org/licenses/by/4.0/).

References Supplementary Table 1

1. Wenning GK, Geser F, Krismer F, Seppi K, Duerr S, Boesch S, et al. The natural history of multiple system atrophy: a prospective European cohort study. Lancet Neurol. 2013;12(3):264-74. DOI: 10.1016/s1474-4422(12)70327-7.
2. Gilman S, Wenning GK, Low PA, Brooks DJ, Mathias CJ, Trojanowski JQ, et al. Second consensus statement on the diagnosis of multiple system atrophy. Neurology. 2008;71(9):670-6. DOI: 10.1212/01.wnl.0000324625.00404.15.
3. Lees AJ, Hardy J, Revesz T. Parkinson's disease. Lancet. 2009;373(9680):2055-66. DOI: 10.1016/s0140-6736(09)60492-x.
4. Wenning GK, Tison F, Seppi K, Sampaio C, Diem A, Yekhlef F, et al. Development and validation of the Unified Multiple System Atrophy Rating Scale (UMSARS). Mov Disord. 2004;19(12):1391-402. DOI: 10.1002/mds.20255.
5. Tomlinson CL, Stowe R, Patel S, Rick C, Gray R, Clarke CE. Systematic review of levodopa dose equivalency reporting in Parkinson's disease. Mov Disord. 2010;25(15):2649-53. DOI: 10.1002/mds.23429.
6. Fanciulli A, Goebel G, Lazzeri G, Scherfler C, Gizewski ER, Granata R, et al. Early distinction of Parkinson-variant multiple system atrophy from Parkinson's disease. Mov Disord. 2019;34(3):440-1. DOI: 10.1002/mds.27635.
7. Goetz CG, Poewe W, Rascol O, Sampaio C, Stebbins GT, Counsell C, et al. Movement Disorder Society Task Force report on the Hoehn and Yahr staging scale: status and recommendations. Mov Disord. 2004;19(9):1020-8. DOI: 10.1002/mds.20213.
8. Freeman R, Wieling W, Axelrod FB, Benditt DG, Benarroch E, Biaggioni I, et al. Consensus statement on the definition of orthostatic hypotension, neurally mediated syncope and the postural tachycardia syndrome. Clin Auton Res. 2011;21(2):69-72. DOI: 10.1007/s10286-011-0119-5.
9. Fanciulli A, Leys F, Falup-Pecurariu C, Thijs R, Wenning GK. Management of Orthostatic Hypotension in Parkinson's Disease. J Parkinsons Dis. 2020;10(s1):S57-s64. DOI: 10.3233/jpd-202036.
10. Fanciulli A, Jordan J, Biaggioni I, Calandra-Buonaura G, Cheshire WP, Cortelli P, et al. Consensus statement on the definition of neurogenic supine hypertension in cardiovascular autonomic failure by the American Autonomic Society (AAS) and the European Federation of Autonomic Societies (EFAS): Endorsed by the European Academy of Neurology (EAN) and the European Society of Hypertension (ESH). Clin Auton Res. 2018;28(4):355-62. DOI: 10.1007/s10286-018-0529-8.
11. Rivasi G, Rafanelli M, Mossello E, Brignole M, Ungar A. Drug-Related Orthostatic Hypotension: Beyond Anti-Hypertensive Medications. Drugs Aging. 2020;37(10):725-38. DOI: 10.1007/s40266-020-00796-5.
12. Tsao CW, Aday AW, Almarzooq ZI, Alonso A, Beaton AZ, Bittencourt MS, et al. Heart Disease and Stroke Statistics-2022 Update: A Report From the American Heart Association. Circulation. 2022;145(8):e153-e639. DOI: 10.1161/cir.0000000000001052.
13. Gibbons CH, Schmidt P, Biaggioni I, Frazier-Mills C, Freeman R, Isaacson S, et al. The recommendations of a consensus panel for the screening, diagnosis, and treatment of neurogenic orthostatic hypotension and associated supine hypertension. J Neurol. 2017;264(8):1567-82. DOI: 10.1007/s00415-016-8375-x.
14. Leys F, Eschlböck S, Campese N, Mahlknecht P, Peball M, Goebel G, et al. Family History for Neurodegeneration in Multiple System Atrophy: Does it Indicate Susceptibility? Mov Disord. 2022. DOI: 10.1002/mds.29202.

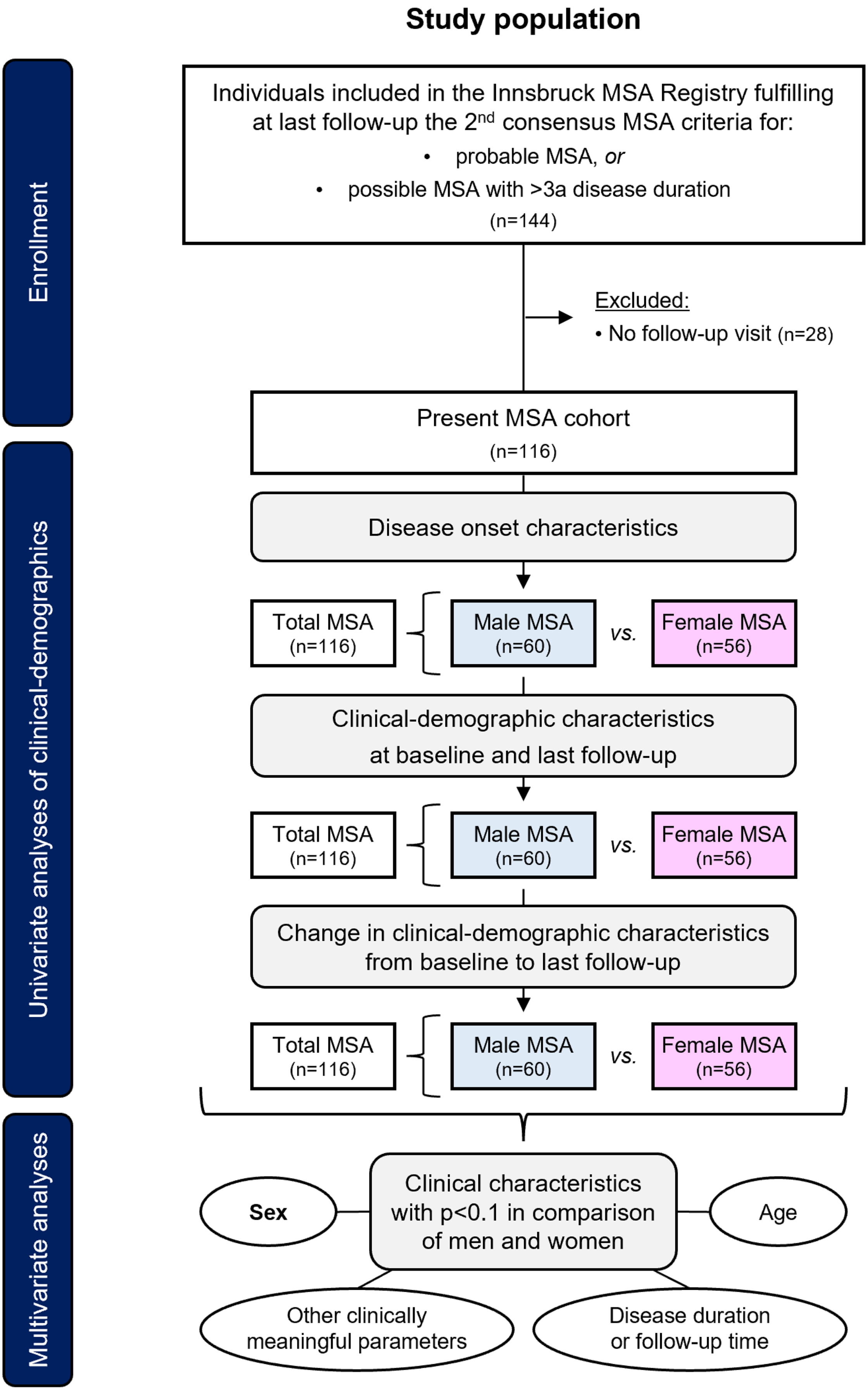

Supplementary Fig. 2 Flow chart and analytic approach of the statistical analysis

Supplementary Table 2 Literature findings with sex-related information in MSA

Publication	Year	Design	Sex-related information: Male sex	Sex-related information: No differences in	Sex-related information: Female sex
Before 1st consensus criteria					
Saito *et al.*[1]	1994	Prospective study including n=59 individuals with olivopontocerebellar atrophy, striatonigral degeneration, or Shy-Drager-Syndrome.[a]		• age at onset;<br>• survival assessed from time of diagnosis or onset.<br>(onset defined by motor or autonomic symptoms, the latter including OH or urinary disturbances)	
Wenning *et al.*[2]	1994	Prospective study including n=100 individuals with probable MSA [3].	• more likely to initially report autonomic symptoms;<br>• longer survival.<br>(onset defined by motor or autonomic symptoms, including erectile/ejaculatory dysfunction in male individuals)	• frequency of urinary urge incontinence and urinary retention requiring catheterization;<br>• survival.<br>(onset defined by motor symptoms)	• associated with better response to L-dopa.
Wenning *et al.*[4]	1995	Retrospective study including n=35 individuals with post-mortem confirmed MSA.	• more frequently showing cerebellar features.	• age at onset or death;<br>• survival.<br>(onset defined by motor or autonomic symptoms, with recording of erectile failure in men)	
Tison *et al.*[5]	1996	Retrospective study including n=100 individuals with probable MSA [3].			• more frequently reporting pain.
Testa *et al.*[6]	1996	Prospective study including n=59 individuals with probable MSA [3].		• survival.<br>(onset defined by motor symptoms)	

Ben-Shlomo *et al.*[7]	1997	Meta-analysis of n=433 individuals with post-mortem confirmed MSA.		• survival.<br>(onset defined by motor or autonomic symptoms, the latter including urinary incontinence, urinary retention, fecal incontinence, or syncope)	
Klockgether *et al.*[8]	1998	Retrospective study including n=67 individuals with clinically diagnosed MSA [9].		• survival.<br>(missing information regarding onset definition)	• faster motor progression as indicated by a shorter time to walking aids or wheelchair dependency.
Between 1st and 2nd consensus criteria					
Testa *et al.*[10]	2001	Prospective study including n=74 individuals with possible or probable MSA [3].		• survival.<br>(onset defined by motor symptoms).	
Watanabe *et al.*[11]	2002	Prospective study including n=230 individuals with probable or definite MSA [12].		• disease progression assessed by ADL milestones corresponding to loss of ability for independent movement;<br>• survival.<br>(onset defined by motor or autonomic symptoms, the latter excluding sexual dysfunction)	
Seppi *et al.*[13]	2005	Prospective study including n=38 individuals with probable MSA-P [12].		• disease progression as indicated by change of MDS-UPDRS Part III.	
Yamamoto *et al.*[14]	2005	Prospective study including n=84 individuals with probable MSA [12].		• abnormal neurogenic change of motor unit potentials as indicated by sphincter EMG.	
Geser *et al.*[15]	2006	Prospective study including n=50 individuals with possible or probable MSA [12].		• disease progression as indicated by change of UMSARS Part I and II.	

Williams *et al.*[16]	2006	Retrospective study including n=91 individuals with post-mortem confirmed MSA.		• frequency of falls.	• higher frequency of fractures.
Tada *et al.*[17]	2007	Retrospective study including n=49 individuals with post-mortem confirmed MSA.		• progression to a bedridden or wheelchair-bound stage;<br>• risk of sudden death;<br>• survival.<br>(onset defined by motor or autonomic symptoms, the latter including postural syncope, OH, or urinary incontinence)	
Schrag *et al.*[18]	2008	Prospective study including n=97 individuals with probable or definite MSA [3].	• borderline longer survival.<br>(missing information regarding applied onset definition)		
O'Sullivan *et al.*[19]	2008	Retrospective study including n=83 individuals with post-mortem confirmed MSA.	• more frequently showing early autonomic dysfunction.		• shorter survival.<br>(onset defined by "the first symptom considered to be attributable to MSA")
After 2nd consensus criteria					
Yamamoto *et al.*[20]	2009	Prospective study including n=256 individuals with possible or probable MSA [12].		• frequency urinary storage and voiding symptoms;<br>• frequency and severity of sexual dysfunction;<br>• quality of life index as indicated by micturition index.	• slightly higher frequency of constipation.
Köllensperger *et al.*[21]	2010	Prospective study including n=437 individuals with possible or probable MSA [12].	• higher frequency of urinary retention.	• frequency of depression, psychotic symptoms, delusions, and dementia;<br>• age at onset.<br>(onset defined by motor or autonomic symptoms)	• higher frequency of urinary incontinence.

Kim *et al.*[22]	2011	Retrospective study including n=455 individuals with probable MSA [9].		• age at onset.<br>(onset defined by motor or autonomic symptoms, the latter including marked urinary incontinence, urinary retention, or postural pre-/syncope)	• longer survival, particularly in individuals with MSA-P.
Wenning *et al.*[23]	2013	Prospective study including n=141 individuals with possible or probable MSA [12].		• sex-ratio among MSA subtypes;<br>• survival.<br>(onset defined by motor or autonomic symptoms, the latter including OH, urge incontinence, or urinary retention)	
Roncevic *et al.*[24]	2014	Retrospective study including n=100 individuals with possible or probable MSA [25].		• survival.<br>(onset defined by motor or autonomic symptoms, the latter excluding sexual dysfunction)	
Yamamoto *et al.*[26]	2014	Retrospective study including n=66 individuals with possible or probable MSA [25].	• more severe voiding symptoms and night-time urinary frequency at baseline.	• motor unit potentials of external anal sphincter EMG.	• better detrusor contraction and lower urethral resistance at baseline;<br>• more severe urinary urgency at follow-up.
Figueroa *et al.*[27]	2014	Retrospective study including n=49 individuals with post-mortem confirmed MSA.		• survival.<br>(onset defined by motor or autonomic symptoms, the latter including OH, urinary incontinence, or retention)	
Low *et al.*[28]	2015	Prospective study including n=175 individuals with probable MSA [12, 25].		• survival.<br>(onset defined by motor or autonomic symptoms, the latter including OH, urinary incontinence, or inability to void)	
Coon *et al.*[29]	2015	Retrospective study including n=685 individuals with possible or probable MSA [25].		• survival.<br>(onset defined by motor or autonomic symptoms, only considering erectile dysfunction when occurring with motor or urinary symptoms)	• longer duration from diagnosis to death.

Starhof *et al.*[30]	2016	Retrospective study including n=115 individuals with probable MSA [25].		• survival.<br>(onset defined by motor or autonomic symptoms)	
Glasmacher *et al.*[31]	2017	Meta-analysis including n=4282 individuals with clinical and post-mortem confirmed MSA.		• survival.<br>(missing information regarding onset definition)	
Savica *et al.*[32]	2017	Retrospective study including n=16 individuals with clinical MSA-P diagnosis [25].		• survival.<br>(onset defined by motor symptoms)	
Zhang *et al.*[33]	2018	Prospective study including n=237 individuals with probable MSA [25].			• associated with depression and anxiety.
Zhang *et al.*[34]	2018	Prospective study including n=131 individuals with probable MSA [25].		• cause of death.	
Jecmenica *et al.*[35]	2018	Prospective study including n=45 individuals with probable MSA-P [12].			• negative predictor of health-related quality of life.
Coon *et al.*[36]	2019	Retrospective study including n=685 individuals with possible or probable MSA [25].	• more likely to initially manifest autonomic-only symptoms;<br>• more likely to develop orthostatic intolerance within one year of onset and require catheterization (overall/within three years of onset);<br>• more severe BP falls on head-up tilt;<br>• higher frequency of sexual dysfunction.	• age at onset;<br>• frequency of MSA subtype;<br>• frequency of parkinsonism, ataxia, falls (at any time), bladder symptoms, stridor, and dream enactment behavior.	• more likely to initially manifest motor symptoms only;<br>• more likely to experience falls within three years of onset;<br>• receive an earlier diagnosis;<br>• longer survival assessed from time of diagnosis or onset.<br>(onset defined by motor or autonomic symptoms, only considering erectile dysfunction when occurring with urinary symptoms)

Foubert-Samier *et al.*[37]	2020	Prospective study including n=261 individuals with possible or probable MSA [25].	• higher supine BP values;<br>• higher systolic BP falls upon standing.	• risk of death;<br>• survival.<br>(onset defined by motor or autonomic symptoms, the latter including OH or neurogenic bladder disturbances)	• faster UMSARS Part I/II progression;<br>• greater disability as indicated by an UMSARS Part IV score ≥3 over the study period;<br>• UMSARS-adjusted: better prognosis.
Cuoco *et al.*[38]	2020	Prospective study including n=55 individuals with possible or probable MSA [25].		• mean L-dopa equivalent daily dosage;<br>• deterioration of executive function and visuo-spatial abilities over time;<br>• other cognitive tests and behavioral items (see publication);<br>• disease duration.<br>(missing information regarding onset definition)	• higher UMSARS Part IV and trend towards higher UMSARS I/II scores;<br>• higher deterioration of attention and motor function (UMSARS) over time;<br>• worse global cognitive status and deficits in attention, planning, language, and visuo-spatial abilities;<br>• borderline more frequent depression.
McCarter *et al.*[39]	2020	Retrospective study including n=182 individuals with possible or probable MSA [25].	• shorter survival.<br>(onset defined by motor or autonomic symptoms, only considering erectile dysfunction when occurring with motor or urinary symptoms)		
Lin *et al.*[40]	2020	Prospective study including n=165 individuals with probable MSA [25].	• associated with excessive daytime sleepiness and REM sleep behavior disorder.		
Gurevich *et al.*[41]	2021	Retrospective study including n=99 individuals with probable MSA [25].	• association of higher supine systolic BP values with increased mortality in MSA-P and borderline association of maximum diastolic orthostatic BP fall with increased mortality in MSA-C;<br>• trend towards longer survival in MSA-P.<br>(onset defined by motor or autonomic symptoms)	• sex-ratio among MSA subtypes.	• borderline association of lower supine systolic BP values with decreased mortality in MSA-P.

Eschlböck *et al.*[42]	2021	Retrospective study including n=74 individuals with possible or probable MSA [25].	• more frequent intake of alpha-blockers;<br>• higher maximum detrusor pressure during micturition and PVR volume.		• higher frequency of urinary incontinence and lower bladder filling volume for first desire to void.
Wang *et al.*[43]	2022	Prospective study including n=66 individuals with probable MSA [25].		• frequency of excessive daytime sleepiness.	
Pavy-LeTraon *et al.*[44]	2022	Prospective study including n=129 individuals with possible or probable MSA [25].	• associated with increased mortality.		
Zhou *et al.*[45]	2022	Retrospective study including n=45 individuals with MSA [25].		• sex-ratio among MSA subtypes;<br>• oculomotor deficits.	
Xie *et al.*[46]	2022	Prospective study including n=70 individuals with probable MSA [25].			• higher frequency of frailty and sarcopenia.
Hu *et al.*[47]	2022	Prospective study including n=61 individuals with possible or probable MSA [25].	• higher frequency of sexual dysfunction;<br>• more pronounced sexual function subdomain scores as indicated by the Non-Motor-Symptom-Scale [48].		
Zhang *et al.*[49]	2023	Retrospective study including n=1492 individuals with probable MSA [25].	• more frequently showing an MSA-C subtype;<br>• higher frequency of OH, drooling, urinary and sexual dysfunction.	• frequency of anhidrosis, depression, anxiety, and cognitive impairment.	• higher frequency of probable REM sleep behavior disorder and constipation.
Altmann *et al.*[50]	2023	Retrospective study including n=110 individuals with probable MSA [25].		• risk of falls.	

Bailey *et al.*[51]	2023	Retrospective study including n=685 individuals with possible or probable MSA [25].	• higher frequency of undergoing procedures to address urinary symptoms;<br>• higher frequency of urinary voiding disturbances.	• frequency of MSA subtype;<br>• frequency of bladder symptoms (overall) as well as of urinary urgency;<br>• frequency of developing urinary before motor symptoms;<br>• time from diagnosis to urinary symptom onset;<br>• the proportion undergoing PVR volume measurement and applied technique;<br>• time from urinary symptom onset to catheterization;<br>• age at onset, years from onset to diagnosis, disease duration.<br>(onset defined by motor or autonomic symptoms, only considering erectile dysfunction when occurring with motor or urinary symptoms)	• higher frequency of urinary incontinence.

Findings are presented in chronological order.

[a] indicates diagnosis according to the diagnostic criteria proposed by the Research Committee of Ataxic Diseases (Ministry of Health and Welfare, Japan, 1992).

ADL, activities of daily living; BP, blood pressure; MDS-UPDRS, Movement Disorder Society – Unified Parkinson's Disease Rating Scale; MSA, multiple system atrophy; MSA-C, cerebellar-variant MSA; MSA-P, Parkinson-variant MSA; OH, orthostatic hypotension; PVR, postvoid residual; UMSARS, Unified MSA Rating Scale.

References Supplementary Table 2

1. Saito Y, Matsuoka Y, Takahashi A, Ohno Y. Survival of patients with multiple system atrophy. Intern Med. 1994;33(6):321-5. DOI: 10.2169/internalmedicine.33.321.
2. Wenning GK, Ben Shlomo Y, Magalhães M, Daniel SE, Quinn NP. Clinical features and natural history of multiple system atrophy. An analysis of 100 cases. Brain. 1994;117(Pt 4):835-45. DOI: 10.1093/brain/117.4.835.
3. Quinn N, Wenning G. Multiple system atrophy. Br J Hosp Med. 1994;51(9):492-4.
4. Wenning GK, Ben-Shlomo Y, Magalhães M, Daniel SE, Quinn NP. Clinicopathological study of 35 cases of multiple system atrophy. J Neurol Neurosurg Psychiatry. 1995;58(2):160-6. DOI: 10.1136/jnnp.58.2.160.
5. Tison F, Wenning GK, Volonte MA, Poewe WR, Henry P, Quinn NP. Pain in multiple system atrophy. J Neurol. 1996;243(2):153-6. DOI: 10.1007/bf02444007.
6. Testa D, Filippini G, Farinotti M, Palazzini E, Caraceni T. Survival in multiple system atrophy: a study of prognostic factors in 59 cases. J Neurol. 1996;243(5):401-4. DOI: 10.1007/bf00868999.
7. Ben-Shlomo Y, Wenning GK, Tison F, Quinn NP. Survival of patients with pathologically proven multiple system atrophy: a meta-analysis. Neurology. 1997;48(2):384-93. DOI: 10.1212/wnl.48.2.384.
8. Klockgether T, Ludtke R, Kramer B, Abele M, Burk K, Schols L, et al. The natural history of degenerative ataxia: a retrospective study in 466 patients. Brain. 1998;121(Pt 4):589-600. DOI: 10.1093/brain/121.4.589.
9. Quinn N. Multiple system atrophy—the nature of the beast. J Neurol Neurosurg Psychiatry. 1989;Suppl:78-89.
10. Testa D, Monza D, Ferrarini M, Soliveri P, Girotti F, Filippini G. Comparison of natural histories of progressive supranuclear palsy and multiple system atrophy. Neurol Sci. 2001;22(3):247-51. DOI: 10.1007/s100720100021.
11. Watanabe H, Saito Y, Terao S, Ando T, Kachi T, Mukai E, et al. Progression and prognosis in multiple system atrophy: an analysis of 230 Japanese patients. Brain. 2002;125(Pt 5):1070-83. DOI: 10.1093/brain/awf117.
12. Gilman S, Low PA, Quinn N, Albanese A, Ben-Shlomo Y, Fowler CJ, et al. Consensus statement on the diagnosis of multiple system atrophy. J Neurol Sci. 1999;163(1):94-8.
13. Seppi K, Yekhlef F, Diem A, Luginger Wolf E, Mueller J, Tison F, et al. Progression of parkinsonism in multiple system atrophy. J Neurol. 2005;252(1):91-6. DOI: 10.1007/s00415-005-0617-2.
14. Yamamoto T, Sakakibara R, Uchiyama T, Liu Z, Ito T, Awa Y, et al. When is Onuf's nucleus involved in multiple system atrophy? A sphincter electromyography study. J Neurol Neurosurg Psychiatry. 2005;76(12):1645-8. DOI: 10.1136/jnnp.2004.061036.
15. Geser F, Wenning GK, Seppi K, Stampfer-Kountchev M, Scherfler C, Sawires M, et al. Progression of multiple system atrophy (MSA): a prospective natural history study by the European MSA Study Group (EMSA SG). Mov Disord. 2006;21(2):179-86. DOI: 10.1002/mds.20678.
16. Williams DR, Watt HC, Lees AJ. Predictors of falls and fractures in bradykinetic rigid syndromes: a retrospective study. J Neurol Neurosurg Psychiatry. 2006;77(4):468-73. DOI: 10.1136/jnnp.2005.074070.
17. Tada M, Onodera O, Ozawa T, Piao YS, Kakita A, Takahashi H, et al. Early development of autonomic dysfunction may predict poor prognosis in patients with multiple system atrophy. Arch Neurol. 2007;64(2):256-60. DOI: 10.1001/archneur.64.2.256.
18. Schrag A, Wenning GK, Quinn N, Ben-Shlomo Y. Survival in multiple system atrophy. Mov Disord. 2008;23(2):294-6. DOI: 10.1002/mds.21839.
19. O'Sullivan SS, Massey LA, Williams DR, Silveira-Moriyama L, Kempster PA, Holton JL, et al. Clinical outcomes of progressive supranuclear palsy and multiple system atrophy. Brain. 2008;131(Pt 5):1362-72. DOI: 10.1093/brain/awn065.

20. Yamamoto T, Sakakibara R, Uchiyama T, Liu Z, Ito T, Awa Y, et al. Questionnaire-based assessment of pelvic organ dysfunction in multiple system atrophy. Mov Disord. 2009;24(7):972-8. DOI: 10.1002/mds.22332.
21. Köllensperger M, Geser F, Ndayisaba JP, Boesch S, Seppi K, Ostergaard K, et al. Presentation, diagnosis, and management of multiple system atrophy in Europe: final analysis of the European multiple system atrophy registry. Mov Disord. 2010;25(15):2604-12. DOI: 10.1002/mds.23192.
22. Kim HJ, Jeon BS, Lee JY, Yun JY. Survival of Korean patients with multiple system atrophy. Mov Disord. 2011;26(5):909-12. DOI: 10.1002/mds.23580.
23. Wenning GK, Geser F, Krismer F, Seppi K, Duerr S, Boesch S, et al. The natural history of multiple system atrophy: a prospective European cohort study. Lancet Neurol. 2013;12(3):264-74. DOI: 10.1016/s1474-4422(12)70327-7.
24. Roncevic D, Palma JA, Martinez J, Goulding N, Norcliffe-Kaufmann L, Kaufmann H. Cerebellar and parkinsonian phenotypes in multiple system atrophy: similarities, differences and survival. J Neural Transm (Vienna). 2014;121(5):507-12. DOI: 10.1007/s00702-013-1133-7.
25. Gilman S, Wenning GK, Low PA, Brooks DJ, Mathias CJ, Trojanowski JQ, et al. Second consensus statement on the diagnosis of multiple system atrophy. Neurology. 2008;71(9):670-6. DOI: 10.1212/01.wnl.0000324625.00404.15.
26. Yamamoto T, Sakakibara R, Uchiyama T, Yamaguchi C, Ohno S, Nomura F, et al. Time-dependent changes and gender differences in urinary dysfunction in patients with multiple system atrophy. Neurourol Urodyn. 2014;33(5):516-23. DOI: 10.1002/nau.22441.
27. Figueroa JJ, Singer W, Parsaik A, Benarroch EE, Ahlskog JE, Fealey RD, et al. Multiple system atrophy: prognostic indicators of survival. Mov Disord. 2014;29(9):1151-7. DOI: 10.1002/mds.25927.
28. Low PA, Reich SG, Jankovic J, Shults CW, Stern MB, Novak P, et al. Natural history of multiple system atrophy in the USA: a prospective cohort study. Lancet Neurol. 2015;14(7):710-9. DOI: 10.1016/S1474-4422(15)00058-7.
29. Coon EA, Sletten DM, Suarez MD, Mandrekar JN, Ahlskog JE, Bower JH, et al. Clinical features and autonomic testing predict survival in multiple system atrophy. Brain. 2015;138(Pt 12):3623-31. DOI: 10.1093/brain/awv274.
30. Starhof C, Korbo L, Lassen CF, Winge K, Friis S. Clinical Features in a Danish Population-Based Cohort of Probable Multiple System Atrophy Patients. Neuroepidemiology. 2016;46(4):261-7. DOI: 10.1159/000444325.
31. Glasmacher SA, Leigh PN, Saha RA. Predictors of survival in progressive supranuclear palsy and multiple system atrophy: a systematic review and meta-analysis. J Neurol Neurosurg Psychiatry. 2017;88(5):402-11. DOI: 10.1136/jnnp-2016-314956.
32. Savica R, Grossardt BR, Bower JH, Ahlskog JE, Boeve BF, Graff-Radford J, et al. Survival and Causes of Death Among People With Clinically Diagnosed Synucleinopathies With Parkinsonism: A Population-Based Study. JAMA Neurol. 2017;74(7):839-46. DOI: 10.1001/jamaneurol.2017.0603.
33. Zhang LY, Cao B, Zou YT, Wei QQ, Ou RW, Zhao B, et al. Depression and anxiety in multiple system atrophy. Acta Neurol Scand. 2018;137(1):33-7. DOI: 10.1111/ane.12804.
34. Zhang L, Cao B, Zou Y, Wei QQ, Ou R, Liu W, et al. Causes of Death in Chinese Patients with Multiple System Atrophy. Aging Dis. 2018;9(1):102-8. DOI: 10.14336/ad.2017.0711.
35. Jecmenica-Lukic MV, Pekmezovic TD, Petrovic IN, Dragasevic NT, Kostić VS. Factors associated with deterioration of health-related quality of life in multiple system atrophy: 1-year follow-up study. Acta Neurol Belg. 2018;118(4):589-95. DOI: 10.1007/s13760-018-0962-4.
36. Coon EA, Nelson RM, Sletten DM, Suarez MD, Ahlskog JE, Benarroch EE, et al. Sex and gender influence symptom manifestation and survival in multiple system atrophy. Auton Neurosci. 2019;219:49-52. DOI: 10.1016/j.autneu.2019.04.002.
37. Foubert-Samier A, Pavy-Le Traon A, Guillet F, Le-Goff M, Helmer C, Tison F, et al. Disease progression and prognostic factors in multiple system atrophy: A prospective cohort study. Neurobiol Dis. 2020;139:104813. DOI: 10.1016/j.nbd.2020.104813.

38. Cuoco S, Picillo M, Cappiello A, Carotenuto I, Erro R, Russillo MC, et al. Effects of gender on cognitive and behavioral manifestations in multiple system atrophy. J Neural Transm (Vienna). 2020;127(6):925-34. DOI: 10.1007/s00702-020-02169-z.
39. McCarter SJ, Coon EA, Savica R, St Louis EK, Bower JH, Benarroch EE, et al. Lower Vitamin B12 Level at Multiple System Atrophy Diagnosis Is Associated With Shorter Survival. Mov Disord. 2020;35(8):1462-6. DOI: 10.1002/mds.28070.
40. Lin JY, Zhang LY, Cao B, Wei QQ, Ou RW, Hou YB, et al. Sleep-related symptoms in multiple system atrophy: determinants and impact on disease severity. Chin Med J (Engl). 2020;134(6):690-8. DOI: 10.1097/cm9.0000000000001211.
41. Gurevich T, Merkin L, Rozenberg A, Fisher A, Atanasova Mishkova-Serafimova E, Klepikov D, et al. Interrelationships between Survival, Sex, and Blood Pressure in Patients with Multiple System Atrophy. Neuroepidemiology. 2021:1-6. DOI: 10.1159/000512697.
42. Eschlböck S, Kiss G, Krismer F, Fanciulli A, Kaindlstorfer C, Raccagni C, et al. Urodynamic Evaluation in Multiple System Atrophy: A Retrospective Cohort Study. Mov Disord Clin Pract. 2021;8(7):1052-60. DOI: 10.1002/mdc3.13307.
43. Wang H, Tang X, Zhou J, Xu Y. Excessive Daytime Sleepiness Is Associated With Non-motor Symptoms of Multiple System Atrophy: A Cross-Sectional Study in China. Front Neurol. 2021;12:798771. DOI: 10.3389/fneur.2021.798771.
44. Pavy-Le Traon A, Foubert-Samier A, Ory-Magne F, Fabbri M, Senard JM, Meissner WG, et al. Ambulatory blood pressure and drug treatment for orthostatic hypotension as predictors of mortality in patients with multiple system atrophy. Eur J Neurol. 2022;29(4):1025-34. DOI: 10.1111/ene.15232.
45. Zhou H, Sun Y, Wie L, Wang X, Jiang Y, Li F, et al. Quantitative assessment of oculomotor function by videonystagmography in multiple system atrophy. Clin Neurophysiol. 2022;141:15-23. DOI: 10.1016/j.clinph.2022.05.019.
46. Xie D, Huang H, Zhao Q, Ning P, Shen Q, Lu H, et al. Prevalence and associated factors of frailty and sarcopenia in multiple system atrophy and progressive supranuclear palsy: a cross-sectional study. Neurol Sci. 2022;43(11):6329-37. DOI: 10.1007/s10072-022-06296-1.
47. Hu WZ, Cao LX, Yin JH, Zhao XS, Piao YS, Gu WH, et al. Non-motor symptoms in multiple system atrophy: A comparative study with Parkinson’s disease and progressive supranuclear palsy. Front Neurol. 2022;13:1081219. DOI: 10.3389/fneur.2022.1081219.
48. Chaudhuri KR, Martinez-Martin P, Brown RG, Sethi K, Stocchi F, Odin P, et al. The metric properties of a novel non-motor symptoms scale for Parkinson’s disease: Results from an international pilot study. Mov Disord. 2007;22(13):1901-11. DOI: 10.1002/mds.21596.
49. Zhang J, Han J, Shi Z, Zhang J, Zhou Z, Liu J, et al. The characteristic of nonmotor symptoms with different phenotypes and onsets in multiple system atrophy patients. J Clin Neurosci. 2023;109:1-5. DOI: 10.1016/j.jocn.2022.12.012.
50. Altmann CF, Koschel J, Jost WH. Predictors of falls in Parkinson’s disease, progressive supranuclear palsy, and multiple system atrophy: a retrospective study. Neurol Neurochir Pol. 2023;57(3):297-304. DOI: 10.5603/PJNNS.a2023.0036.
51. Bailey ES, Hooshmand SJ, Badihian N, Sandroni P, Benarroch EE, Bower JH, et al. Sex and Gender Influence Urinary Symptoms and Management in Multiple System Atrophy. J Mov Disord. 2023;16(2):196-201. DOI: 10.14802/jmd.23016.

Supplementary Table 3 Disease onset characteristics of male and female individuals included in the Innsbruck MSA Registry

	**Total MSA cohort** (n=116)	**Male MSA** (n=60)	**Female MSA** (n=56)	***p*** (M *vs.* F)
Sociodemographic data				
Age at disease onset, years	58.3 [51.3; 65.0]	61.6 [52.9; 66.2]	56.4 [49.9; 62.5]	**0.019**
Initial clinical feature(s)				
Motor	100 (86.2)	50 (83.3)	50 (89.3)	0.353
Parkinsonian symptoms	64 (55.2)	34 (56.7)	30 (53.6)	0.738
Cerebellar symptoms	40 (34.5)	18 (30.0)	22 (39.3)	0.293
Autonomic (n=58 M, 55 F)	25 (22.1)	15 (25.9)	10 (18.2)	0.326
Orthostatic intolerance	9 (7.8)	7 (11.7)	2 (3.6)	0.165
Bladder disturbances (n=58 M, 55 F)	19 (16.8)	10 (17.2)	9 (16.4)	0.901
Motor & autonomic (n=58 M, 55 F)	9 (8.0)	5 (8.6)	4 (7.3)	1.000

Bold font indicates statistically significant results.
F, female; M, male; MSA, multiple system atrophy; n, number.

Supplementary Table 4 Clinical characteristics of Innsbruck MSA individuals showing no association with female or male sex in the multivariable analysis

Bold font indicates statistically significant results. Other covariates included in the multivariable analysis and showing a significant positive association with the outcome variable are indicated by [a], those with a significant negative association by [b]. OR are summarized by number (95% CI).

**Outcome variable** (i.e., clinical characteristic showing an association with female or male sex at univariate analysis)	**M/F**	**Covariate sex** OR (95% CI)	***p***	**Other covariates in the multivariable models**
At baseline visit				
UMSARS Part IV score, (>2 yes *vs.* no)	F	3.1 (0.8-11.4)	0.094	At baseline visit: age; **disease duration[a]**; comorbidities (cardiovascular disease; **diabetes mellitus[a]**); **MSA subtype (Parkinson-variant[a])**.
Intake of CNS-active drugs with BP lowering side effects (yes *vs.* no)	F	11.0 (0.7-175.7)	0.089	At baseline visit: age; disease duration; UMSARS Part IV score; **depression[a]**; speaking/acting out dreams.
At last available follow-up				
Rigidity (yes *vs.* no)	F	1.1 (0.1-17.0)	0.951	At last follow-up: age; disease duration; **Hoehn & Yahr stage[a]**.
Dopaminergic response ("no or poor" yes *vs.* no)	M	2.0 (0.6-6.6)	0.231	At last follow-up: **age[a]**; disease duration; Hoehn & Yahr stage; LEDD.
History of orthostatic syncope (yes *vs.* no)	M	3.1 (1.0-9.6)	0.055	At last follow-up: age; disease duration; LEDD; intake of CNS-active drugs with BP lowering side effects; antihypertensive medication intake.
Catheterization (yes *vs.* no)	M	2.1 (0.9-4.6)	0.073	At last follow-up: age; disease duration; comorbidities (diabetes mellitus).

continues

Cardiovascular disease (yes *vs.* no)	M	2.1 (0.8-5.5)	0.154	At last follow-up: **age[a]**; disease duration; severe OH; supine hypertension; comorbidities (diabetes mellitus).
Change from baseline to last available follow-up				
Parkinsonism (new onset yes *vs.* no)	F	1.0 (0.2-7.2)	0.970	Initial clinical feature: **cerebellar symptoms[a]**. Age at last follow-up; follow-up time. Adjustment from baseline to follow-up of: LEDD.
Bradykinesia (new onset yes *vs.* no)	F	1.4 (0.2-9.2)	0.718	Initial clinical feature: **cerebellar symptoms[a]**. Age at last follow-up; follow-up time. Adjustment from baseline to follow-up of: LEDD.
Rigidity (new onset yes *vs.* no)	F	1.6 (0.2-10.8)	0.638	Initial clinical feature: **cerebellar symptoms[a]**. Age at last follow-up; follow-up time. Adjustment from baseline to follow-up of: LEDD.
Postural abnormalities (new onset yes *vs.* no)	F	2.0 (0.6-7.2)	0.284	Age at last follow-up; follow-up time. Worsening/adjustment from baseline to follow-up of: **dopaminergic response[a]**; LEDD.
Increase of LEDD (≥300 mg/day yes *vs.* no)	F	1.6 (0.4-5.7)	0.478	Age at last follow-up; follow-up time. Worsening/new onset from baseline to follow-up of: dopaminergic response; severe OH.
Dopaminergic response (worsening yes *vs.* no)	M	1.8 (0.5-5.7)	0.345	Initial clinical feature: parkinsonian symptoms. **Age at last follow-up[a]**; follow-up time. Adjustment from baseline to follow-up of: LEDD.
Supine hypertension (new onset *vs.* no)	M	2.9 (0.9-9.1)	0.066	Age at last follow-up; follow-up time. New start/onset from baseline to follow-up of: OH treatment (pressor agents); comorbidities (cardiovascular disease).

For the purpose of the multivariable binary regression analysis, variables were categorized as follows:

- UMSARS Part IV score at baseline visit, >2: in 49% (n=18/37) women *vs.* 26% (n=11/43) men (*p*=0.039).
- dopaminergic response at last follow-up, "no or poor": in 81% (n=39/48) men *vs.* 62% (n=31/50) women (*p*=0.035).
- increase in LEDD from baseline to last follow-up, ≥300 mg/day: in 59% (n=27/46) women *vs.* 35% (n=16/46) men (*p*=0.022).

BP, blood pressure; CI, confidence interval; F, female; LEDD, L-dopa equivalent daily dosage; M, male; MSA; multiple system atrophy; OH, orthostatic hypotension; OR, odds ratio; UMSARS, Unified MSA Rating Scale.

STrengthening the **R**eporting of **OB**servational studies in **E**pidemiology (STROBE) **Statement** version 4

	Item No	Recommendation	
**Title and abstract**	1	(*a*) Indicate the study's design with a commonly used term in the title or the abstract.	✓
		(*b*) Provide in the abstract an informative and balanced summary of what was done and what was found.	✓
**Introduction**			
Background/rationale	2	Explain the scientific background and rationale for the investigation being reported.	✓
Objectives	3	State specific objectives, including any prespecified hypotheses.	✓
**Methods**			
Study design	4	Present key elements of study design early in the paper.	✓
Setting	5	Describe the setting, locations, and relevant dates, including periods of recruitment, exposure, follow-up, and data collection.	✓
Participants	6	(*a*) *Cohort study*—Give the eligibility criteria, and the sources and methods of selection of participants. Describe methods of follow-up.	✓
		(*b*) *Cohort study*—For matched studies, give matching criteria and number of exposed and unexposed.	NA
Variables	7	Clearly define all outcomes, exposures, predictors, potential confounders, and effect modifiers. Give diagnostic criteria, if applicable.	✓ (if appli-cable)
Data sources/ measurement	8	For each variable of interest, give sources of data and details of methods of assessment (measurement).	✓
Bias	9	Describe any efforts to address potential sources of bias.	✓
Study size	10	Explain how the study size was arrived at.	✓
Quantitative variables	11	Explain how quantitative variables were handled in the analyses. If applicable, describe which groupings were chosen and why.	✓

Statistical methods	12	(*a*) Describe all statistical methods, including those used to control for confounding.	✓
		(*b*) Describe any methods used to examine subgroups and interactions.	✓
		(*c*) Explain how missing data were addressed.	✓
		(*d*) *Cohort study*—If applicable, explain how loss to follow-up was addressed.	NA
		(*e*) Describe any sensitivity analyses.	NA
**Results**			
Participants	13	(a) Report numbers of individuals at each stage of study—e.g., numbers potentially eligible, examined for eligibility, confirmed eligible, included in the study, completing follow-up, and analysed.	✓
		(b) Give reasons for non-participation at each stage.	✓
		(c) Consider use of a flow diagram.	✓
Descriptive data	14	(a) Give characteristics of study participants (e.g., demographic, clinical, social) and information on exposures & potential confounders.	✓
		(b) Indicate number of participants with missing data for each variable of interest.	✓
		(c) *Cohort study*—Summarise follow-up time (e.g., average and total amount).	✓
Outcome data	15	*Cohort study*—Report numbers of outcome events or summary measures over time.	✓
Main results	16	(*a*) Give unadjusted estimates and, if applicable, confounder-adjusted estimates and their precision (e.g., 95% confidence interval). Make clear which confounders were adjusted for and why they were included.	✓
		(*b*) Report category boundaries when continuous variables were categorized.	✓
		(*c*) If relevant, consider translating estimates of relative risk into absolute risk for a meaningful time period.	NA
Other analyses	17	Report other analyses done—e.g., analyses of subgroups and interactions, and sensitivity analyses.	✓ (if applicable)

**Discussion**			
Key results	18	Summarise key results with reference to study objectives.	✓
Limitations	19	Discuss limitations of the study, taking into account sources of potential bias or imprecision. Discuss both direction and magnitude of any potential bias.	✓
Interpretation	20	Give a cautious overall interpretation of results considering objectives, limitations, multiplicity of analyses, results from similar studies, and other relevant evidence.	✓
Generalisability	21	Discuss the generalisability (external validity) of the study results.	✓
**Other information**			
Funding	22	Give the source of funding and the role of the funders for the present study and, if applicable, for the original study on which the present article is based.	✓

Information on the STROBE Initiative is available at www.strobe-statement.org.
